# State‐specific Regulation of Electrical Stimulation in the Intralaminar Thalamus of Macaque Monkeys: Network and Transcriptional Insights into Arousal

**DOI:** 10.1002/advs.202402718

**Published:** 2024-06-27

**Authors:** Zhao Zhang, Yichun Huang, Xiaoyu Chen, Jiahui Li, Yi Yang, Longbao Lv, Jianhong Wang, Meiyun Wang, Yingwei Wang, Zheng Wang

**Affiliations:** ^1^ Department of Anesthesiology Huashan Hospital, Fudan University 12 Urumqi Middle Rd, Jing'an District Shanghai 200040 China; ^2^ School of Psychological and Cognitive Sciences Beijing Key Laboratory of Behavior and Mental Health State Key Laboratory of General Artificial Intelligence IDG/McGovern Institute for Brain Research Peking‐Tsinghua Center for Life Sciences Peking University 5 Yiheyuan Rd, Haidian District Beijing 100871 China; ^3^ Institute of Natural Sciences and School of Mathematical Sciences Shanghai Jiao Tong University 800 Dongchuan RD, Minhang District Shanghai 200240 China; ^4^ Department of Neurosurgery Brain Computer Interface Transition Research Center Beijing Tiantan Hospital Capital Medical University 119 South Fourth Ring Rd West, Fengtai District Beijing 100070 China; ^5^ National Resource Center for Non‐Human Primates Kunming Primate Research Center National Research Facility for Phenotypic & Genetic Analysis of Model Animals (Primate Facility) Kunming Institute of Zoology Chinese Academy of Sciences 32 East of Jiaochang Rd Kunming Yunnan 650223 China; ^6^ Department of Medical Imaging Henan Provincial People's Hospital & the People's Hospital of Zhengzhou University No. 7 Weiwu Road Zhengzhou Henan 450003 China; ^7^ School of Biomedical Engineering Hainan University 58 Renmin Avenue Haikou Hainan 570228 China

**Keywords:** arousal, electrical stimulation, intralaminar thalamus, MRI, transcriptomics

## Abstract

Long‐range thalamocortical communication is central to anesthesia‐induced loss of consciousness and its reversal. However, isolating the specific neural networks connecting thalamic nuclei with various cortical regions for state‐specific anesthesia regulation is challenging, with the biological underpinnings still largely unknown. Here, simultaneous electroencephalogram‐fuctional magnetic resonance imaging (EEG‐fMRI) and deep brain stimulation are applied to the intralaminar thalamus in macaques under finely‐tuned propofol anesthesia. This approach led to the identification of an intralaminar‐driven network responsible for rapid arousal during slow‐wave oscillations. A network‐based RNA‐sequencing analysis is conducted of region‐, layer‐, and cell‐specific gene expression data from independent transcriptomic atlases and identifies 2489 genes preferentially expressed within this arousal network, notably enriched in potassium channels and excitatory, parvalbumin‐expressing neurons, and oligodendrocytes. Comparison with human RNA‐sequencing data highlights conserved molecular and cellular architectures that enable the matching of homologous genes, protein interactions, and cell types across primates, providing novel insight into network‐focused transcriptional signatures of arousal.

## Graphical abstract

This study identifies an ILN‐driven arousal network responsible for rapid arousal during anesthesia through simultaneous EEG‐fMRI and DBS in macaques. Network‐based RNA sequencing reveals genes preferentially expressed within this arousal network, notably enriched in potassium channels and excitatory, parvalbumin‐expressing neurons, and oligodendrocytes. Comparative analysis with human data reveals conserved molecular and cellular architectures, offering insights into transcriptional signatures of arousal.

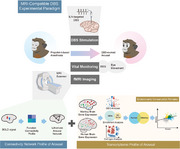

## 1 Introduction

Anesthetics have been widely demonstrated to induce graded levels of anesthesia ranging from mild sedation to deep coma, often characterized by behavioral, electroencephalography (EEG), and neuroimaging markers.^[^ ^1^, ^2^, ^3^ ^]^ This spectrum of anesthetic states is believed to be governed by a complex interplay of genetic factors,^[^ ^3^, ^4^, ^5^ ^]^ yet the specifics of this genetic landscape remain largely uncharted. While most studies have concentrated on a narrow selection of genes within varied anesthetic contexts, the broader genetic interconnections influencing arousal and anesthesia are not well‐understood. A critical question arises: Do gene expression patterns exhibit uniformity across spatially separated but functionally interconnected brain regions upon arousal from anesthesia? This inquiry is pivotal in understanding the genetic underpinnings of arousal from anesthesia.^[^ ^5^ ^]^ Emerging evidence from heritability studies and imaging‐genomics/transcriptomics points to the role of common genetic variations in structuring and harmonizing multiple resting‐state networks in the human neocortex.^[^ ^6^, ^7^, ^8^ ^]^ Furthermore, the pattern of network‐biased gene expression, consistent across limbic and somato/motor networks in both cortical and subcortical structures, is suggested to be conserved in non‐human primates.^[^ ^9^ ^]^ This consistency underscores the potential of high‐throughput network‐level transcriptomic analyses in identifying molecular markers critical for network functionality in both health and pathological states.^[^ ^10^, ^11^ ^]^ In the context of arousal, the distinctive enrichment of specific genes within the identified network, as opposed to their expression in the broader brain, could be indicative of the preferential involvement of certain cell types in arousal. It may also point to an increased responsiveness to specific neurotransmitters or a unique, layer‐specific expression of genes linked to arousal. Unraveling these patterns offers the potential for unprecedented molecular and cellular insights into the biological mechanisms underpinning arousal.^[^ ^12^ ^]^ Despite significant advancements in delineating the structural and functional frameworks of arousal networks, our understanding of their molecular genetic correlates remains limited.

Extensive research in both humans and animals highlights the crucial role of long‐range thalamocortical and corticocortical interactions in managing anesthetic‐induced alterations in consciousness.^[^ ^2^, ^13^ ^]^ Despite this, the exact neural mechanisms dictating anesthesia and consciousness levels remain under debate.^[^ ^13^, ^14^ ^]^ The thalamus, particularly its intralaminar nuclei (ILN), is a focal point of interest due to its complex interactions with the cerebral cortex and brainstem.^[^ ^15^, ^16^, ^17^ ^]^ The ILN is uniquely positioned, establishing extensive connections with various cortical areas and receiving inputs from the basal forebrain and brainstem arousal systems.^[^ ^16^, ^18^ ^]^ Deep brain stimulation (DBS) targeting the ILN has enriched our understanding of its role in sensory processing, consciousness, and pain modulation.^[^ ^19^, ^20^, ^21^, ^22^ ^]^ Recent breakthroughs in stimulating the central, rather than ventrolateral, thalamus in monkeys have demonstrated the activation of extensive thalamocortical and frontoparietal networks, reflecting a variety of brain states.^[^ ^23^, ^24^ ^]^ The potential of ILN stimulation in therapeutic applications for brain disorders is significant, yet the outcomes are variable and heavily dependent on the specific ILN target locations and the underlying neurological or psychiatric conditions.^[^ ^25^, ^26^ ^]^ Nevertheless, the precise causal impact of ILN on state‐dependent brain regulation remains an area for further exploration.^[^ ^27^, ^28^ ^]^

**Scheme 1:**
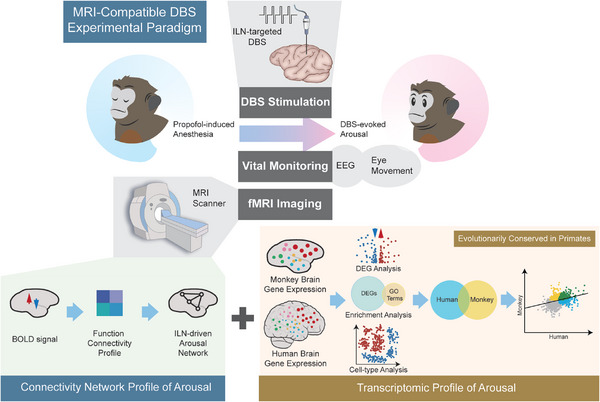
A scheme of the experimental strategy, data generation, and analysis. Our experimental strategy involved a multi‐step approach. The initial phase involved simultaneous EEG‐fMRI and intralaminar thalamus‐targeted DBS in monkeys under the influence of propofol anesthesia. This allowed for an in‐depth examination of the neural networks associated with arousal. Subsequently, we performed transcriptomic profiling of these identified networks in the macaque model. To broaden the current understanding, we compared these profiles with corresponding human data, providing a comprehensive view of the convergent and divergent aspects of arousal mechanisms across primates.

Towards these aims, we focused on defining a state‐specific arousal regulatory network through the combined use of EEG‐fuctional magnetic resonance imaging (fMRI) and DBS of ILN in macaques under stringently controlled propofol anesthesia. Building on this, we conducted network‐based RNA‐sequencing analysis, leveraging our recently developed transcriptomics atlas of the macaque brain, aligned with MRI coordinates.^[^ ^29^ ^]^ Additionally, we scrutinized layer‐specific and cell type‐specific gene expression within this arousal network, utilizing independent transcriptomic datasets from macaque brains.^[^ ^30^, ^31^ ^]^ A key aspect of our study involved comparing the arousal network‐associated gene expression profiles in macaques with those in humans, using data from the Allen Human Brain Atlas (AHBA).^[^ ^32^ ^]^ This research marks a pioneering foray into integrating arousal network mapping with brain stimulation and transcriptomic profiling in nonhuman primates, as depicted in **Scheme** **1**. It establishes an innovative, multi‐scale, and cross‐species framework for examining the transcriptomic bases of arousal and consciousness.

## 2 Results

### 2.1 Physiological and Behavioral Effects of ILN‐DBS Modulation

**Figure 1:**
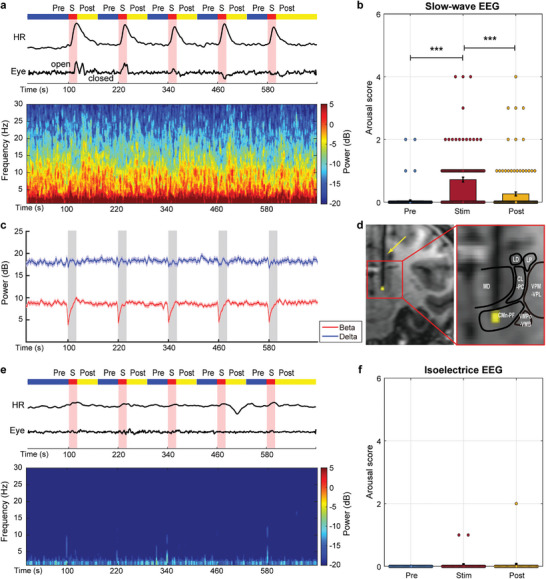
Physiological and behavioral effects of ILN‐DBS during finely tuned propofol anesthesia. a) Traces of heartbeat and behavioral recordings (upper panel) and spectrogram of the left frontal electrode (lower panel) during EEG slow‐wave oscillations in a typical ILN‐DBS run. b) Population mean arousal scores (±SEM) before, during, and after stimulation in monkeys under EEG slow‐wave oscillations (*n =* 125). Circles stand for individual stimulation events. *** *p <* 0.001, Tukey‐Kramer multiple comparison test. c) Population averaged band power throughout a typical ILN‐DBS run under EEG slow‐wave oscillations. The solid line indicates the mean power averaged across runs, whereas the shaded regions represent ±SEM. d) Stimulation sites in monkey M‐J. Coronal section of the right hemisphere is 8.5 mm anterior to the interaural line of the F99 template (left panel). The yellow point indicates the electrode tip. The arrow indicates the electrode. Thalamic subareas labeled with reference to the SARM atlas (right panel): CL‐PC, central lateral and paracentralis nuclei, anterior section of ILN; CMn‐PF, centromedian‐parafasicularis complex, posterior section of ILN; LD, lateral dorsal thalamus; LP, lateral posterior thalamus; MD, mediodorsal thalamus; VMPo‐VMB, ventromedial posterior+basal thalamus; VPM‐VPL, ventroposterior medial and lateral thalamus. e) Traces of heartbeat and behavioral recordings (upper panel) and spectrogram of the left frontal electrode (lower panel) during EEG isoelectric lines in a typical ILN‐DBS run. f) Population mean arousal scores (±SEM) before, during, and after stimulation in monkeys under EEG isoelectric lines (*n =* 50). Circles stand for individual stimulation events.

We performed a series of DBS experiments in monkeys targeting the centromedian‐parafascicular (CMn‐PF) complex, part of the ILN (illustrated in **Figure** **1**d), under graded levels of anesthesia after comparing the functional connectivity (FC) profiles of different states (Figures S1 and S2, Supporting Information, section A and B). First, we assessed changes in arousal scores similarly reported in previous studies^[^ ^33^, ^34^ ^]^ and EEG signals before, during, and after stimulations when the slow‐wave oscillations were readily visible in the EEG recordings.^[^ ^1^, ^2^ ^]^ We monitored 75 blocks of low‐current (200 µA) stimulation and observed that electrical stimulation of the ILN significantly increased the arousal level (Figure 1a,b, ***p < 0.001, Tukey‐Kramer multiple comparison test). Behavioral changes were time‐locked to stimulation periods (Figure 1a, upper panel), including spontaneous blinks or continuous eyes‐open, oral movement, and a sharp increase in heart rate (HR) followed by a gradual return to baseline. Meanwhile, one example EEG spectrogram recorded from the left frontal electrode (Figure 1a, lower panel) showed marked power changes (like the beta band) in response to each stimulation event, consistent with the change in group averaged EEG power trace (Figure 1c). These behavioral and EEG changes were observed in both subjects (M‐J and M‐G, Figure S3a, Supporting Information, ** *p <* 0.01, *** *p <* 0.001, Tukey‐Kramer multiple comparison test).

We also tested high‐current (400 µA) stimulation using the same paradigm. Group analysis of 40 high‐current stimulation blocks revealed significant arousal effects by ILN stimulation (Figure S3b, Supporting Information, right panel, ** *p <* 0.01, *** *p <* 0.001, Tukey‐Kramer multiple comparison test), stronger and long‐lasting compared to the low‐current condition. As demonstrated in the spectrograms, electrical stimulation evoked prominent power changes in the delta and beta bands (Figure S3b, Supporting Information, left panel). Note that delta power inhibition and enhancement of high‐frequency activities are often used to indicate arousal from anesthesia.^[^ ^1^ ^]^ Furthermore, we repeated the ILN‐DBS stimulation in both subjects in the propofol‐induced deeper anesthesia state, in which the EEG displayed a continuous isoelectric line. We collected 50 blocks of low‐ and high‐current stimulation (30 low‐ and 20 high‐current blocks) and found that neither changes in arousal score nor EEG signal were elicited by ILN‐DBS stimulation (Figure 1e,f).

### 2.2 ILN‐DBS Selectively Modulated a Set of Brain Regions

**Figure 2:**
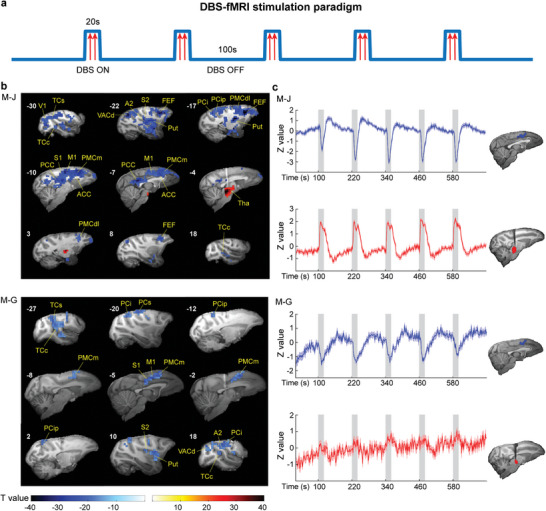
Spatial and temporal profiles of brain response to ILN‐DBS during slow‐wave oscillations. a) An example block‐design fMRI paradigm with DBS. Stimulation to the right ILN was delivered during 20 s ON blocks followed by 100 s OFF blocks. Each fMRI run consisted of five ON and six OFF blocks. b) Averaged activation map of M‐J and M‐G. BOLD responses evoked by ILN‐DBS were overlaid onto individual T1 images. Elevated regions are shown in red, and inhibited regions are shown in blue (*p <* 10^−7^, FWE corrected). c) Mean time courses of significantly modulated clusters in the cortex (blue) and near the stimulation site (red) exhibit robust negative and positive BOLD responses in both subjects. A2, secondary auditory cortex; ACC, anterior cingulate cortex; FEF, frontal eye field; M1, primary motor cortex; PCi, inferior parietal cortex; PCip, intraparietal cortex; PCs, superior parietal cortex; PCC, posterior cingulate cortex; PMCdl, dorsolateral premotor cortex; PMCm, medial premotor cortex; Put, putamen; S1, primary somatosensory cortex; S2, secondary somatosensory cortex; TCc, central temporal cortex; TCs, superior temporal cortex; Tha, thalamus; V1, visual area 1 (primary visual cortex); VACd, anterior visual area (dorsal part).

We customized an experimental setup that allowed concurrent DBS and fMRI acquisition in macaques, and we aimed to map the functional activity evoked by ILN‐DBS in each subject with finely tuned propofol anesthesia by contrasting DBS‐on and DBS‐off conditions (**Figure** **2**a). At the single‐subject level, evoked blood oxygen‐level dependent (BOLD) activation of M‐J (Figure 2b, upper panel; *p <* 10^−7^, familywise error rate (FWE) corrected) was prominent around the stimulation site, whereas deactivation of BOLD signal evoked by ILN‐DBS was widely distributed in frontal, parietal, cingulate, temporal, and occipital lobes and the striatum, including the frontal eye field (FEF), primary motor cortex (M1), dorsolateral premotor cortex (PMCdl), medial premotor cortex (PMCm), primary somatosensory cortex (S1), secondary somatosensory cortex (S2), intraparietal cortex (PCip), inferior parietal cortex (PCi), secondary auditory cortex (A2), superior temporal cortex (TCs), central temporal cortex (TCc), anterior visual area (VAC), primary visual cortex (V1), anterior/ posterior cingulate cortex (ACC/PCC), and putamen (Put). Both activation and deactivation evoked by ILN‐DBS were replicated in Subject M‐G. A set of brain regions, including M1, PMCm, S1, S2, PCip, PCi, superior parietal cortex, A2, TCs, TCc, VAC, and Put, were deactivated (Figure 2b, lower panel; *p <* 10^−7^, FWE corrected), whereas relatively weaker activation was observed around the stimulation site (Figure 2c, right panel, red regions of M‐G; *p <* 0.05, FWE corrected). To better visualize the temporal response pattern of activation and deactivation, we extracted the mean time course of the BOLD response from those significantly modulated clusters near the stimulation site (activation) and the largest cortical clusters overlapped in both subjects (deactivation) and plotted them for each subject (Figure 2c, red stands for activation and blue for deactivation).

### 2.3 Functional Connectivity Profiles and Dynamics of the ILN‐Driven Arousal Network

**Figure 3:**
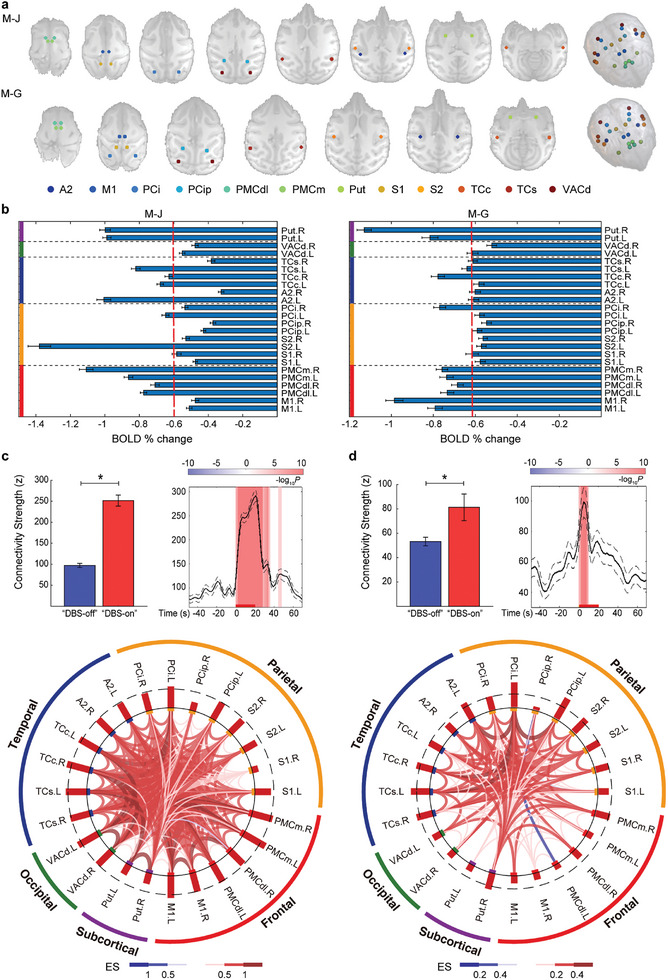
FC profiles and dynamics of ILN‐driven arousal network. a) Co‐activated regions of both monkeys were determined as regions of interest bilaterally (ROIs, radius = 2 mm) to constitute an ILN‐driven arousal network. b) Event‐related percentage change in BOLD signal (BOLD% change) of DBS‐on versus DBS‐off epochs. ILN‐DBS resulted in robust and consistent modulation of PMC, PCi, TCs, TCc, and Put in both subjects. The dashed red lines label the top 50% of nodes with significant BOLD% change values. The vertical solid lines on the left indicate the corresponding lobe of the nodes, i.e., frontal lobe (red), parietal lobe (orange), temporal lobe (blue), occipital lobe (green), and striatum (purple). c,d) Effects of ILN‐DBS on dynamic connectivity within the ILN‐driven arousal network of M‐J c) and M‐G d). Connectivity strength (CS) shows a significant increase during the “DBS‐on” condition (*p <* 0.001; upper left panel). The time course of CS is plotted with solid and dashed lines as mean±SEM (upper right panel). The stimulation onset is aligned to 0 s, and the stimulation period is marked in red from 0 to 20 s (10 TRs). Time points with significantly increased or decreased (*p <* 0.001, two‐sample *t*‐test) CS relative to “DBS‐off” are highlighted in red or blue, respectively. The color bar indicates the *P* values. Altered functional connections with node information are plotted in the bottom panel. Red or blue lines present significantly increased or decreased connections, respectively (*p <* 0.05, NBS correction with edge‐wise *p <* 0.001), with width and color indicating effect size (Hedges’ g value). For nodes, red and blue bars in the interlayer indicate the effect sizes (Hedges’ g value) of increased and decreased node strength, respectively. The dashed line labels the top 50% of nodes with significantly altered node strength.

We thus identified a consensus ILN‐DBS response map from two subjects, hereinafter referred to as the ILN‐driven arousal network, which included a set of 24 regions spanning over bilateral M1, PMCdl, PMCm, S1, S2, PCip, PCi, A2, TCc, TCs, dorsal part of VAC (VACd), and Put, as shown in **Figure** **3**a. Figure 3b shows the percentage change in BOLD signals (BOLD% change) in these regions comparing DBS‐on with DBS‐off periods. Based on these 24 identified regions, we next constructed a functional connectivity network and deployed a dynamic FC analysis on each subject, which was applied to characterize the temporal changes in FC during ILN‐DBS. A robust increase in the connectivity strength of ILN‐DBS‐induced dynamic FCs was observed in both subjects and was mainly locked in the stimulation periods (Figure 3c,d, upper panels; *p <* 0.001). Note that the potentiation effect of M‐J was stronger and broader with a longer duration relative to M‐G, which corresponded to a stronger arousal effect (Figure S3a, Supporting Information). The FC profiles within this network exhibited a consistent elevated response (indicated by the effect size of changes in FC) to ILN‐DBS (Figure 3c,d, lower panels; *p <* 0.05, NBS correction with edgewise *p <* 0.001), and the interlayer in the plots showed an upregulated effect on the node strength of 24 regions. Notably, we implemented stringent control of confounding factors such as head motion (regressing out covariates including six head motion parameters, white matter, and ventricular signal) and confirmed the above observation (Figure S4a–d, Supporting Information). Evidently, a robust increase in FC and node strength of PMC, PCip, PCi, and TCs involving multisensory processing was consistently observed in both subjects.

### 2.4 Gene Expression and Enrichment Analysis of the ILN‐Driven Arousal Network

**Figure 4:**
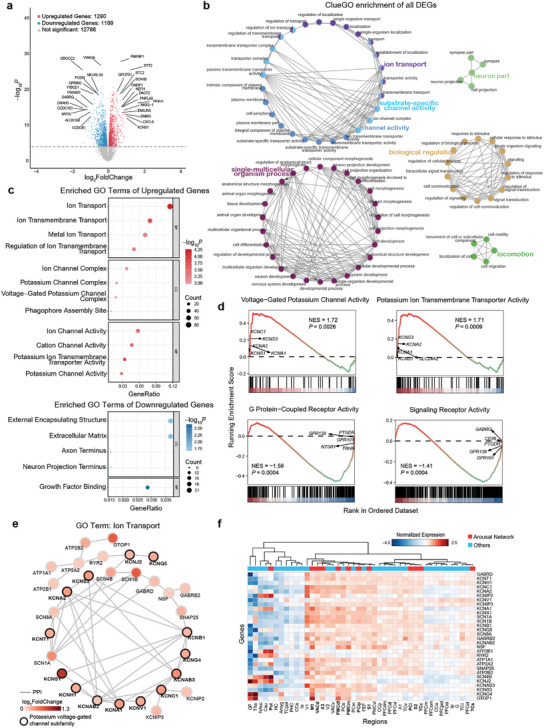
Functional enrichment of genes differentially expressed in the ILN‐driven arousal network. a) Volcano plot of DEGs within the ILN‐driven arousal network relative to the rest of the brain. In the total of 15 275 genes, 1290 genes were upregulated (red) and 1199 genes were downregulated (blue) in the arousal network (*p <* 10^−4^, FDR corrected). The dotted line represents *P* = 10^−4^. b) ClueGO network visualization of GO functional enrichment for all 2489 DEGs. Only GO terms with *p <* 10^−4^ (hypergeometric test, FDR corrected) are shown. The connectivity of the GO term network is described by nodes that are shared between the genes with a kappa score over 0.5. Each node represents a GO term, and each color represents a GO group. The bold fonts indicate the most important functional GO terms that define the names of each group. c) The bubble chart represents the GO functional enrichment for upregulated genes (upper panel) and downregulated genes (lower panel). The top four statistically significant terms for each category are shown (*p <* 0.05, hypergeometric test, FDR corrected). The bubble size represents the number of overlapping genes between the gene list and each GO term. The color bar represents the *P* value. d) The GSEA of GO MF between the arousal network and the rest of the brain. Four significant enriched terms are shown (|NES| > 1.4, *p <* 0.01, GSEA permutation test). The top 5 genes are annotated for each term. e) PPI network of the “ion transport” GO term in upregulated genes. The largest subclusters in the PPI network of the GO term are presented. The nodes’ color represents the signedlog_2_FoldChange of the DEG analysis between the arousal network and the rest of the brain, and edges denote direct PPIs. Genes encoding potassium voltage‐gated channel subfamily proteins are indicated with a black outline. f) Heatmap of the normalized expression for genes in Figure 4e among brain regions (listed in Table S3, Supporting Information for details). Regions are hierarchically clustered using Euclidean distance dissimilarity. Those regions that belong to the arousal network are annotated in red. GO, gene ontology; BP, biological process; CC, cellular component; MF, molecular function; PPI, protein‐protein interaction. NES, normalized enrichment score.

Subsequently, we utilized a comprehensive Cynomolgus Macaque Brain Atlas (CMBA), which included RNA‐sequencing data from 878 samples spanning 110 regions in the macaque brain and aimed to identify genes preferentially expressed within the ILN‐driven arousal network relative to the rest of the brain using differential expression gene (DEG) analysis. After preprocessing the regional metadata, we assigned 294 samples to the arousal network (525 samples to the rest of the brain) and retained 15 275 genes for DEG analysis. Then, we identified 2489 genes (1290 upregulated and 1199 downregulated) that were differentially expressed in the arousal network and compared effect‐size changes (log_2_FoldChange) with the corresponding brain signature (**Figure** **4**a; *p <* 10^−4^, false discovery rate (FDR) corrected).

To understand the functional organization of these DEGs, we first performed a ClueGO analysis (Figure 4b). The results showed that several clusters have interrelated processes. Notably, three ion channel‐related clusters– “ion transport”, “substrate‐specific channel activity”, and “channel activity”– are highly interconnected both within and between the clusters. This underscores the critical involvement of ion channel mechanisms in the arousal network. We next conducted gene ontology (GO) enrichment analysis for the upregulated genes and downregulated genes (Figure 4c). Interestingly, the upregulated gene sets were highly enriched in ion channel‐related terms, including “ion transport” (*P* = 5.43 × 10^−5^, hypergeometric test, FDR corrected for all comparisons below), “ion transmembrane transport” (*P* = 1.62 × 10^−4^) for biological process (BP); “ion channel complex” (*P* = 0.001), “potassium channel complex” (*P* = 0.001), and “voltage‐gated potassium channel complex” (*P* = 0.001) for cellular component (CC); and “ion channel activity” (*P* = 3.27 × 10^−4^), “cation channel activity” (*P* = 3.27 × 10^−4^), “potassium ion transmembrane transporter activity” (*P* = 1.14 × 10^−4^), and “potassium channel activity” (*P* = 3.27 × 10^−4^) for molecular function (MF). Evidently, the potassium channel‐related terms were strikingly enriched in the upregulated genes. Downregulated genes were enriched in neuron projection‐ and signaling binding‐related annotation terms, such as “axon terminus” (*P* = 0.016) and “neuron projection terminus” (*P* = 0.021) for CC and “growth factor binding” (*P* = 0.002) for MF. In addition, gene set enrichment analysis (GSEA) was performed to validate the above findings based on the fold change of all genes (Figure 4d). The results showed significantly increased expression of genes typically presented in potassium channel‐related terms, including “voltage‐gated potassium channel activity” (normalized enrichment score (NES) = 1.72, *P* = 0.0026, GSEA permutation test) and “potassium ion transmembrane transporter activity” (NES = 1.71, *P* = 0.0009), consistent with GO enrichment analyses.

We further conducted a protein‐protein interaction (PPI) analysis with these upregulated genes using the String database^[^ ^35^ ^]^ and displayed the main cluster within the “ion transport” term in Figure 4e. The results showed that the interaction network was mainly composed of the voltage‐gated potassium (Kv) channel subunits (such as KCNC1, KCNAB2, KCNA1, KCNAB3, and KCNS3), the Kv channel interacting proteins (including KCNIP2 and KCNIP3), and several subunits of voltage‐gated sodium channels such as SCN8A. Among these genes, KCNS1 exhibited the highest degree of quantitative change in the arousal network (log_2_FoldChange = 1.05). Normalized expression of the genes presented in Figure 4e is shown as a heatmap in Figure 4f, indicating elevated expression patterns within the arousal network relative to the rest of the brain, particularly in regions involving sensorimotor and multisensory processing (e.g., S1, M1, PMC, and PCip).

### 2.5 Cell‐Type and Laminar Analysis of Arousal Network‐Associated DEGs

**Figure 5:**
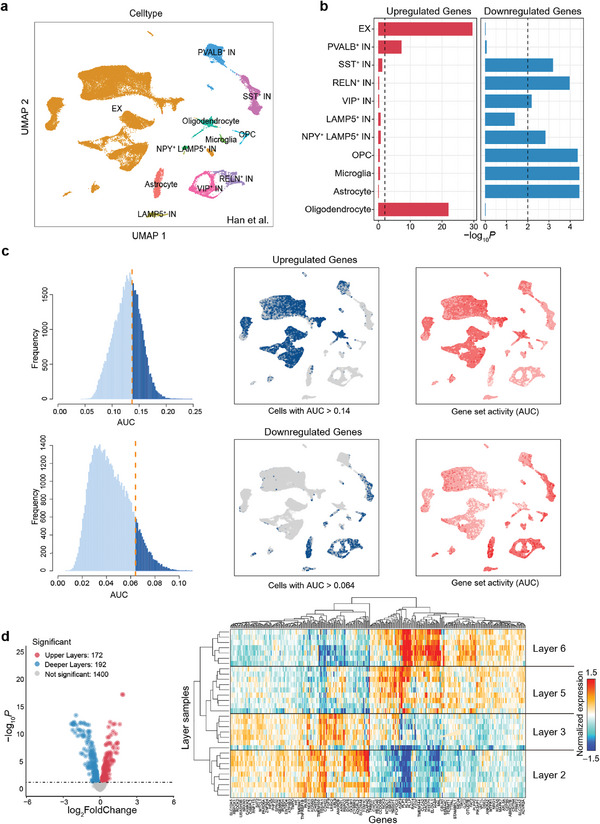
Cell type enrichment and layer‐specific expression of genes differentially expressed in the arousal network. a) UMAP visualization of 11 cell‐type clusters identified in the neocortex of Macaca fascicularis using snRNA‐seq data from Han et al. The classification of the single nucleus with each color represents a predefined cell type. b) Bar plots depict the cell‐type enrichment from snRNA‐seq for upregulated genes and downregulated genes. Cell types with *p <* 0.01 (Fisher's exact test, FDR corrected) were considered to be significant. The dotted line represents *P* = 0.01. c) Cell‐level enrichment of upregulated genes and downregulated genes using AUCell.AUC histogram (left panels) depicts cell frequency of calculating the AUC scores in upregulated genes (upper) and downregulated genes (lower). The AUC threshold was set as 0.14 for upregulated genes and 0.064 for downregulated genes. The UMAP plot was colored with cells that have an AUC score above the threshold (middle panels). UMAP plots based on the AUC score of each cell (right panels). EX, PAVLB^+^ IN, and oligodendrocytes exhibit higher AUC scores for upregulated genes (upper), while SST^+^ IN, RELN^+^ IN, VIP^+^ IN, NPY^+^ LAMP5^+^ IN, astrocytes, OPCs, and microglia exhibit higher AUC scores for downregulated genes (lower). d) Layer‐specific expression in the arousal network DEGs. Volcano plot of layer‐specific expressed genes between the cortical layers 2/3 and 5/6 among DEGs in the arousal network using the cortical microarray data from Bernard et al (left panel). Of these 2489 DEGs, 1764 genes were identifiable in the microarray data. A total of 172 out of 1764 genes were highly expressed in layer 2/3, and 192 genes were highly expressed in layer 5/6 (*p <* 0.05, FDR corrected). The dotted line indicates *P* = 0.05. A hierarchically clustered heatmap of these layer‐specific expressed genes across different cortical layers is shown in the right panel. The heatmap was labeled with a gene name in every fourth column, and a complete list of all these genes is given in Table S4, Supporting Information. EX, excitatory neurons; IN, inhibitory neurons; OPCs, oligodendrocyte precursor cells.

These observations motivated us to perform cell‐type and laminar RNA‐seq analyses to test whether the arousal network‐biased gene set was enriched for specific cell types and cortical layers. Using single‐nucleus RNA‐sequencing (snRNA‐seq) data from the monkey brain,^[^ ^30^ ^]^ we conducted uniform manifold approximation and projection (UMAP) and clustering analyses and identified 11 cell types (**Figure** **5**a), including excitatory neurons (EX), 6 subtypes of inhibitory neurons (IN, i.e., SST^+^, PVALB^+^, NPY^+^, LAMP5^+^, RELN^+^, VIP^+^), microglia, astrocytes, oligodendrocytes and oligodendrocyte precursor cells (OPCs). Subsequently, these cell type annotations were applied to the enrichment analysis of the upregulated and downregulated genes in the arousal network (Figure 5b). Upregulated genes were significantly enriched in EX (*P* = 3.40 × 10^−30^, Fisher's exact test, FDR corrected for all comparisons below), PVALB^+^ IN (*P* = 7.22 × 10^−8^), and oligodendrocytes (*P* = 1.19 × 10^−22^). Meanwhile, downregulated genes were significantly expressed in SST^+^ IN (*P* = 6.67 × 10^−4^), RELN^+^ IN (*P* = 1.09 × 10^−4^), VIP^+^ IN (*P* = 6.81 × 10^−3^), NPY^+^ LAMP5^+^ IN (*P* = 1.51 × 10^−3^), OPCs (*P* = 4.65 × 10^−5^), microglia (*P* = 3.84 × 10^−5^), and astrocytes (*P* = 3.84 × 10^−5^). Additionally, similar enrichments were obtained by using a cell‐level enrichment method called AUCell (Figure 5c). The UMAP plots based on the “area under the curve” (AUC) score of each cell showed that EX, PAVLB^+^ IN, and oligodendrocytes exhibit higher AUC scores for upregulated genes (upper panel), and SST^+^ IN, RELN^+^ IN, VIP^+^ IN, NPY^+^ LAMP5^+^ IN, astrocytes, OPCs, and microglia exhibit higher AUC scores for downregulated genes (lower panel). These results consistently revealed that upregulated genes were mainly enriched in excitatory neurons and PVALB subtype inhibitory neurons, while downregulated genes were mainly enriched in other subtypes of inhibitory neurons and most glial cells. Furthermore, we validated the present results using two other publicly available snRNA‐seq datasets (Figure S6a–d, Supporting Information).

To investigate whether these identified DEGs of the arousal network are specifically expressed in deeper cortical layers that receive feedback signals from superficial layers and preferentially project major cortico‐thalamic afferents to the ILN, we analyzed the cortical microarray data covering the dorsolateral prefrontal cortex and temporal cortex from Bernard et al.^[^ ^31^ ^]^ Among the 2489 DEGs, 1764 genes were identified from the microarray data, and expression data from layers 2, 3, 5, and 6 were available to apply linear models to test for expression differences.^[^ ^9^ ^]^ As a result, we found 172 genes highly expressed in layer 2/3 and 192 genes highly expressed in layer 5/6 (Figure 5d, left panel; *p <* 0.05, FDR corrected; listed in Table S4, Supporting Information). This indicates that these interlayer differential genes were not highly expressed within a particular layer but were widely distributed among Layer 2/3 and Layer 5/6 (shown as a heatmap in Figure 5d, right panel).

### 2.6 Conservation of Gene Expression in the Human Brain

To investigate whether arousal network‐biased gene expression is conserved in the human brain, we analyzed human gene expression data from AHBA.^[^ ^32^ ^]^ We first parcellated the monkey and human brains with the Regional Map atlas^[^ ^36^ ^]^ (see Table S3, Supporting Information), as we have done before,^[^ ^37^, ^38^ ^]^ to facilitate cross‐species comparison. Then we performed a one‐to‐one mapping of the 24 regions identified in the monkeys to the human brain to construct the arousal network in the human brain and repeated DEG analysis. We found 1123 upregulated and 513 downregulated genes among 15 633 genes obtained from AHBA (*p <* 10^−4^, FDR corrected).

**Figure 6:**
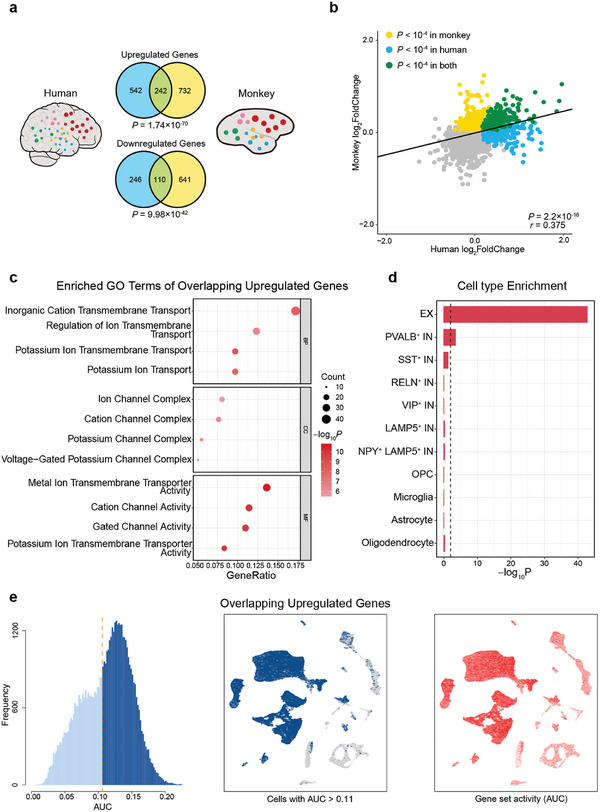
Overlapping upregulated genes in the arousal network conserved across primate species. a) Venn diagrams of the number of overlapping upregulated and downregulated genes in the arousal network between humans and monkeys. Among the homologous genes retained in the two species, 242 homologous genes (30.87% in humans) were upregulated (upper panel) and 110 genes (30.90% in humans) were downregulated (lower panel) in both humans and monkeys, which significantly exceeds the overlap expected by chance (*p <* 0.001, hypergeometric test). b) Gene‐wise log_2_FoldChange in the monkey arousal network was positively correlated to log_2_FoldChange in the human one (r  =  0.312, *p <* 0.001). Yellow dots represent the upregulated genes only in monkey data, the blue dots represent those only in human data, and the green dots represent the overlapping in both. c) The bubble chart represents the GO functional enrichment for overlapping upregulated genes. The top four statistically significant terms for each category are shown (*p <* 0.05, hypergeometric test, FDR corrected). The bubble size represents the number of overlapping genes between the gene list and each GO term. The color bar represents the *P* value. d) Bar plot depicting the cell‐type enrichment from snRNA‐seq for overlapping upregulated genes. The dotted line represents *P* = 0.01. Cell types with *p <* 0.01 (Fisher's exact test, FDR corrected) were considered to be significant. e) Cell‐level enrichment of overlapping upregulated genes using AUCell. The AUC histogram (left panel) depicts the cell frequency of calculating the AUC scores in overlapping upregulated genes. The AUC threshold was set as 0.11. The UMAP plot is colored with cells that have an AUC score above the threshold (middle panel). UMAP plots based on the AUC score of each cell (right panel). EX, PAVLB^+^ IN, and oligodendrocytes exhibit higher AUC scores for these genes.

Combining 15 633 genes from AHBA and 15 275 genes from CMBA, 10 252 homologous genes between humans and monkeys were retained using the homologene package (https://github.com/oganm/homologene) in R. Among these homologous genes, 784 upregulated genes and 356 downregulated genes were retained in humans, while 974 upregulated genes and 751 downregulated genes were retained in monkeys. Notably, in both humans and monkeys, 242 homologous genes (30.87% in humans) were upregulated, and 110 genes (30.90% in humans) were downregulated, exceeding the overlap expected by chance (**Figure** **6**a; *p <* 0.001, hypergeometric test). The gene fold changes calculated in the monkey's arousal network were significantly correlated with those in humans (Figure 6b; *r*  =  0.375, *P* < 0.001, Pearson's correlation), indicating a consistent expression pattern within the arousal network between the two species.

The results of GO enrichment analyses on the 242 overlapping upregulated genes obtained above‐revealed enrichment in ion channel‐related terms (Figure 6c), such as “regulation of ion transmembrane transport” (*P* = 4.24 × 10^−7^, hypergeometric test, FDR corrected for all comparisons below), “potassium ion transmembrane transport” (*P* = 2.25 × 10^−9^), and “potassium ion transport” (*P* = 8.80 × 10^−9^) for BP; “ion channel complex” (*P* = 3.10 × 10^−6^), “potassium channel complex” (*P* = 4.45 × 10^−7^), and “voltage‐gated potassium channel complex” (*P* = 4.45 × 10^−7^) for CC; and “potassium ion transmembrane transporter activity” (*P* = 1.32 × 10^−10^) for MF, in line with the results of monkeys (Figure 4c, upper panel). In addition, both cell enrichment analysis (Figure 6d) and AUCell analysis (Figure 6e) confirmed the specific enrichment of these overlapping upregulated genes in EX (*P* = 1.72 × 10^−43^, Fisher's exact test, FDR corrected for all comparisons below) and PVALB^+^ IN (*P* = 3.15 × 10^−4^), which was consistent with the results of monkeys (Figure 5b,c). These results together indicate that the cellular and molecular patterns of gene expression within the ILN‐driven arousal network are remarkably conserved.

## 3 Discussion

In our study, we developed an MRI‐compatible DBS setup in a propofol‐induced monkey model to investigate the ILN role in regulating anesthesia levels. Our EEG and behavioral signal measurements revealed that ILN electrical stimulation elicited strong, consistent, time‐locked responses to stimuli, effectively transitioning anesthetized monkeys from unconsciousness to wakefulness during slow‐wave oscillations, corroborating recent findings.^[^ ^24^, ^33^, ^34^ ^]^ Slow‐wave oscillations reflecting the slow variation of the resting membrane potential of cortical neurons that switch between depolarized and hyperpolarized states are believed to originate in the cortex and contribute to the cortico‐thalamo‐cortical rhythm.^[^ ^27^, ^39^ ^]^ We observed that the FC profile of the ILN during slow‐wave oscillations predominantly involved multisensory and sensorimotor integration regions such as M1, PMC, S1, and Pcip,^[^ ^40^ ^]^ but was markedly reduced during isoelectric phases (see Supporting Information, section A). This suggests that ILN stimulation enhances information transfer and integration, facilitating cortical communication necessary for wakefulness, multisensory and sensorimotor integration, and directed attention.^[^ ^15^, ^18^ ^]^ However, under deeper anesthesia levels, where EEG may display long‐lasting isoelectric lines,^[^ ^33^ ^]^ neither low‐ or high‐current ILN‐DBS evoked significant behavioral or EEG changes. This could be due to insufficient cortical neuronal firing or lack of suprathreshold depolarization needed for effective cortico‐thalamo‐cortical loop communication.^[^ ^41^ ^]^ As there existed visible differences in BOLD responses and functional connectivity profiles evoked by ILN‐DBS between M‐J and M‐G, we have developed a consensus map of two subjects, highlighting those regions consistently activated to minimize interindividual differences. These findings underscore the importance of multisensory and sensorimotor integration areas like PMC and PCip in consciousness assessment in clinical settings,^[^ ^42^ ^]^ often evaluated through verbal questions such as “what is your name?”, “where are you?”, “open your mouth or hold your hand please?”.

One of the major unexpected findings in our study was the widespread negative BOLD response to ILN‐DBS, accompanied by stimulation‐locked, reproducible arousal behaviors (Figure 1a,b). While the positive BOLD response elicited by electrical stimulation has been well described in previous studies,^[^ ^24^, ^43^, ^44^, ^45^, ^46^ ^]^ the negative BOLD response has often been overlooked.^[^ ^24^, ^45^, ^46^ ^]^ A plausible mechanism for the negative BOLD response in distant brain areas is the activation of inhibitory corticocortical projection neurons^[^ ^44^ ^]^ or downstream polysynaptic stimulation of inhibitory cortical afferents,^[^ ^47^ ^]^ which modulate cortical excitatory networks. Notably, our gene enrichment results support the idea that genes of the ILN‐driven arousal network are enriched in excitatory cells and parvalbumin‐positive (PVALB^+^) inhibitory interneurons. A recent DBS study on Parkinson's patients suggests that the deactivation of BOLD signals may reflect different functional pathways.^[^ ^48^ ^]^ Despite the local suppression of BOLD signals in a set of regions, ILN‐DBS enhanced functional connectivity between them, potentially promoting information transfer and integration, leading to arousal. However, the underlying neurobiological mechanisms of the negative BOLD response warrant future investigation using electrophysiological, immunohistochemical, and pharmacological techniques.

While significant strides have been made in understanding the diverse functions of thalamic nuclei and subnetworks,^[^ ^17^ ^]^ the molecular and cellular basis of the ILN‐driven arousal network remains largely unexplored. Our integrative analysis connects cell type‐, layer‐, and region‐specific molecular characteristics to macroscale connectivity and system‐level functions. We identified network‐specific gene sets in brain areas activated by ILN stimulation, notably finding a significant enrichment in genes related to voltage‐gated potassium (Kv) channels. Animal studies have linked mutations in Kv channel genes, such as Kcna2 in mice,^[^ ^49^ ^]^ to significant alterations in sleep‐wake patterns.^[^ ^3^, ^50^ ^]^ Furthermore, our analysis highlighted a preferential expression of excitatory neurons, PVALB^+^ interneurons, and oligodendrocytes within this arousal network. This aligns with previous findings that fast potassium channels, like Kv3‐type (e.g., KCNC1), are prevalent in PVALB^+^ GABAergic interneurons.^[^ ^3^, ^51^ ^]^ Intralaminar nuclei are rich in PVALB^+^ cells,^[^ ^52^ ^]^ which are thought to modulate excitatory neurons, facilitating short activation times and network oscillations.^[^ ^53^ ^]^ This complements the function of Kv channels, which activate during depolarization and are involved in the rapid repolarization of action potentials, thereby facilitating high‐frequency firing and narrow action potentials.^[^ ^54^ ^]^ The co‐expression of PVALB^+^ IN with oligodendrocyte‐related genes may indicate the involvement of myelinated projection neurons or a specific myelination pattern of PVALB^+^ interneurons,^[^ ^55^ ^]^ potentially crucial for thalamic regulation of synchronized cortical activity during arousal.

The enrichment of Kv channel‐related genes and PVALB^+^ interneurons in the arousal network, as revealed by our study, proposes a novel mechanism for arousal: these elements may enhance rapid repolarization of excitatory neurons, leading to recurrent oscillatory firing and increased network synchrony.^[^ ^37^ ^]^ This aligns with previous insights that ion channel genes are key to modulating functional connectivity and synchrony in brain networks.^[^ ^9^, ^12^ ^]^ However, our findings of gene enrichment across various cortical layers call for a more detailed examination of the roles of calbindin‐positive and parvalbumin‐positive cells within the intralaminar group in arousal and consciousness, potentially adding to the understanding of intracolumnar signaling in consciousness recovery.^[^ ^33^ ^]^

While the evolutionary parallels between macaques and humans are well‐acknowledged in various contexts, direct research into the network effects and molecular/cellular mechanisms of arousal regulation across primate species has been scarce.^[^ ^39^, ^56^, ^57^, ^58^ ^]^ Our study leverages unique transcriptomic resources for both macaque^[^ ^29^ ^]^ and human brains,^[^ ^32^ ^]^ complemented by extensive snRNA‐seq data, enabling a comprehensive comparison of molecular profiles, cell types, and expression patterns between corresponding brain structures in both species. Notably, about 30.87% of the genes upregulated in macaques were also elevated in analogous human brain regions, with a significant correlation in the log_2_FoldChange between species. This suggests a striking conservation of molecular pathways, particularly those involving ion channels, which were predominantly expressed in excitatory and PVALB^+^ neurons in primates. These findings mark a significant advancement in understanding the genetic architecture underlying anesthesia, wakefulness, and arousal.^[^ ^3^ ^]^ The observed prominence of potassium channel activity, known to be influenced by general anesthetics,^[^ ^50^ ^]^ underscores the potential central role of ion channels in anesthetic‐induced unconsciousness and its reversal.^[^ ^5^, ^12^ ^]^ Additionally, our analysis reveals a detailed transcriptomic landscape, offering insights into the cellular composition and gene network dynamics associated with arousal regulation. This could pave the way for new therapeutic strategies targeting the intralaminar thalamus, particularly in repurposing or discovering drugs that modulate the identified genes, pathways, and molecules.^[^ ^26^ ^]^

This study has several limitations that should be considered when interpreting the findings. Firstly, rapid arousal was deliberately induced by electrical stimulation of the ILN, analogous to pharmacologically‐induced reversal of unconsciousness.^[^ ^59^ ^]^ This method may not necessarily correspond with the action mechanisms of anesthetics or reflect normal physiological conditions. Additionally, arousal indicated by behaviors like eye blinks is not equivalent to full consciousness, though the present setting offers an opportunity to dissect distinct components of consciousness.^[^ ^60^ ^]^ Secondly, we observed a mixture of positive and negative BOLD signals in response to ILN stimulation across extensive cortical and subcortical regions. This bipolar response profile warrants further investigation, particularly in relation to enhanced functional synchronization within the arousal network. Our network‐based transcriptomics analyses revealed an enrichment of Kv channel‐related genes and PVALB^+^ interneurons, providing a molecular and cellular basis for subsequent experiments to examine the inhibitory influences on BOLD signals. Thirdly, the transcriptomic findings were derived from the arousal network activated by ILN electrical stimulation, which may be particularly susceptible to inhibitory effects. However, the precise mechanisms by which it counteracts the GABAergic‐promoting action of propofol in the brain remain to be elucidated.

In conclusion, our holistic analysis underscores the pivotal role of potassium channels in excitatory and PVALB^+^ cells within the ILN‐driven network, crucial for counteracting propofol anesthesia. Their involvement in multisensory and sensorimotor integration and brain region synchronization exemplifies how molecular and network aspects collaboratively regulate arousal. This discovery lays the groundwork for innovative therapies that mimic ILN stimulation by targeting Kv channels and excitatory and PVALB^+^ cells. Beyond anesthesia, this study opens new paths for exploring how cellular and molecular dynamics underlie a range of brain functions, including cognitive processes like attention and decision‐making.

## 4 Experimental Section

All experimental procedures were approved by the Animal Care and Use Committee of the Center for Excellence in Brain Science and Intelligence Technology, Chinese Academy of Sciences (ION‐2019009R05), and conformed to National Institutes of Health guidelines for the humane care and use of laboratory animals. Eight monkeys (Macaca fascicularis, 4 male, 3.5–9 years old, 3–7 kg body weight) participated in the resting‐state fMRI (RS‐fMRI) experiment to characterize the FC profiles of the ILN (see Supporting Information, sections A and B). Two monkeys (M‐J and M‐G; Macaca mulatta, male, 11 years old, 11–13 kg body weight) participated in DBS experiments.

### Animal Preparation

Details of animal handling procedures were similarly described in the previous work^[^ ^11^, ^37^, ^38^, ^61^, ^62^, ^63^, ^64^ ^]^ and were briefly summarized here. For all the RS‐fMRI and DBS experiments, propofol (Beijing Fresenius Kabi Pharma Co., Ltd.) served as the sole anesthetic to achieve both induction and maintenance of anesthesia. All subjects were pretrained for indwelling needle placement in the saphenous vein with lidocaine topical anesthesia, and induction of anesthesia was achieved by intravenous injection of propofol (8–10 mg k^−1^ g). Atropine sulfate (0.05 mg k^−1^ g, Shanghai Harvest Pharma Co., Ltd.) was given intramuscularly as premedication to decrease bronchial and salivary secretions. After intubation, subjects were supplemented with 50% oxygen and kept breathing spontaneously during preparation. The infusion rate of propofol was adjusted within a narrow range based on continuously monitored physiological parameters in individuals, including oxygen saturation (SPO_2_), electrocardiogram (ECG), noninvasive blood pressure, rectal temperature (Small Animal Instruments, Inc., Stony Brook, New York), breath rate and end‐tidal CO_2_ (ETCO_2_, Smiths Medical ASD Inc., Dublin, Ohio). SPO_2_ was kept over 95% and rectal temperature was kept constant using a heated water blanket (Gaymar Industries Inc., Orchard Park, New York).

### MRI‐Guided Electrode Positioning

A Cilux chamber (Crist Instruments) was implanted in the parietal bones of each subject for the DBS experiment, providing access to the ILN in the right hemisphere. Craniotomy was performed inside the chamber with the dura intact. Ceramic skull screws and dental acrylic were used to affix the chamber. Surgery was performed under general anesthesia, which was induced by intramuscular injection of ketamine (10 mg k^−1^g; Gutian Pharma Co, Ltd.) and maintained by isoflurane (1%–3%; Lunan Pharma Co, Ltd.). Monopolar platinum/iridium electrodes (50–100 kΩ, FHC Co.Ltd, USA) were used for deep brain stimulation in monkeys and a two‐step MRI‐guided procedure was conducted to precisely localize the electrode inside the brain: 1) After assembling the chamber with a Cilux grid (Crist Instruments), manganese chloride as a contrast agent was injected into the chamber to label the positioning hole of the grid on MRI images. A high‐resolution T1‐weighted anatomical image was subsequently acquired (TR = 2550 ms; TE = 3.94 ms; TI = 900 ms; flip angle = 9°; slices = 128; 0.3 × 0.3 mm^2^ in plane resolution; slice thickness = 0.6 mm). Using this anatomical image and the Subcortical Atlas of the Rhesus Macaque (SARM)^[^ ^65^ ^]^ as a reference, the coordinates of the right ILN were calculated and the targeted positioning hole of the grid was determined. 2) The DBS electrode held by a custom 3D‐printed holder was driven through the targeted hole of the grid by an electric stepping motor. When the electrode was placed into the targeted area, another anatomical image was acquired to calculate the current location of the electrode tip (TR = 2300 ms; TE = 3.43 ms; TI = 800 ms; flip angle = 9°; slices = 144; 0.5 × 0.5 × 0.5 mm^3^). The electrode was iteratively repositioned with reference to updated MRI images until the electrode tip completely reached the targeted location.

### Stimulation Paradigm

The electrical stimulation was delivered using a pulse generator (Master‐8; AMPI) and a current isolator (A365; WPI), which generated biphasic square pulses at 200 Hz with 200 µs pulse width, 200 µA (low‐current stimulation) or 400 µA (high‐current stimulation) amplitude, and cathodal leading. Note that the stimulation parameters were experimentally determined in a pilot study (Figure S5, Supporting Information). Each DBS run consisted of six DBS‐off (100 s) periods with five DBS‐on (20 s) period intervals that lasted 700 s (see Figure 2a). See Table S2, Supporting Information for a detailed description of the datasets of DBS experiments.

### Arousal Scoring

A DBS experiment was conducted in anesthetized monkeys outside of the scanner and evaluated the stimulus‐induced behavioral changes with EEG recording and arousal scoring. An arousal scale modified from the versions used in clinical patients and rhesus macaques^[^ ^33^ ^]^ was adopted here to measure the behavioral effects evoked by ILN stimulation. Five main indicators were included: 1) face movements (0 = none; 1 = small movements; 2 = full frowning/nose twitching); 2) eye movements (0 = none; 1 = eyelid flutters/small blinks; 2 = full eye‐opening with occasional blinks); 3) oral movements (0 = none; 1 = small mouth/jaw/tongue movements; 2 = full jaw movements with multiple repetitions); 4) limb/body movements (0 = none; 1 = small limb/torso movements; 2 = full reach or withdrawal/torso movements); and 5) vital signs (0 = no change, i.e., difference of < 5% breath rate (BR)/heart rate (HR); 1 = difference of > 5% BR/HR; 2 = difference of > 10% BR/HR; compared to 50 s prestimulation baseline). The final arousal score was the sum of these five indicators, ranging from 0 to 10.

### Arousal State Analyses

A typical stimulation block consisted of a DBS‐on period and adjacent pre‐ and post‐stimulation periods. The arousal score during each period was evaluated by a clinical anesthesiologist with the assistance of two experienced experimenters. The difference in HR that was extracted from the ECG electrode using an open‐source Toolbox for Analyzing Heart Rate Variability (HRVTool; https://marcusvollmer.github.io/HRV) was compared across conditions. A sliding window containing 10 RR intervals (the time elapsed between two successive R waves of the QRS signal in ECG) with 1 RR interval step was used, and 60 divided by the mean RR interval from each window was calculated to quantify the dynamic changes in HR. The peak dynamic HR within each period was extracted to represent HR changes. Repeated measures ANOVA was applied to assess group differences in arousal score, using the Tukey‐Kramer method for the correction of multiple comparisons.

### EEG‐fMRI Data Acquisition

The procedure of simultaneous EEG‐fMRI data acquisition was similar to the previous work.^[^ ^38^, ^66^ ^]^ To ensure EEG data quality, the monkey scalp was thoroughly cleaned with abrasive gel and alcohol swabs after hair removal. A customized EEG cap made of stretchable materials was fitted tightly around the monkey's scalp. To facilitate the offline removal of cardio‐ballistic artifacts, an ECG electrode was attached to the back of the monkey (close to the position of the heart). To increase the signal‐to‐noise ratio, the electrode impedance was kept below 5 kΩ by conductive gel filling. After setting up the EEG cap, subjects were restrained within a water blanket in a Sphinx‐like position with the head protruding and facing forward and then transferred to the scanner room. Subjects were kept breathing spontaneously with 50% oxygen during the DBS‐fMRI experiment.

Simultaneous EEG scalp recordings were acquired with BrainVision Recorder software using sintered Ag/AgCl ring electrodes, a BrainAmp MR amplifier, and a 28‐channel EEG cap customized for monkeys (Brain Products GmbH, Gilching, Germany). EEG signals from active electrodes were sampled at 5000 Hz with a resolution of 0.5 mV per bit and a range of ±16 mV. The recording clocks of the MRI console and EEG recording system were synchronized using SyncBox (Brain Products GmbH, Gilching, Germany) for offline artifact removal in EEG signals.

DBS‐fMRI data was acquired using a customized 8‐channel transmit/receive surface coil. Functional images were acquired using a gradient‐echo‐planar imaging sequence (TR = 2000 ms; TE = 28 ms; flip angle = 77°; FOV = 128 mm × 128 mm; 2 mm isotropic resolution; slices = 26). A customized program using LabVIEW (https://www.ni.com/zh‐cn/shop/labview.html) was applied to synchronize electrical stimulation delivery and fMRI scanning, with 350 functional volumes in each DBS‐fMRI run. Anatomical images were acquired using an MPRAGE T1‐weighted sequence (TR = 2300 ms; TE = 3.43 ms; TI = 800 ms; flip angle = 9°; 0.5 mm isotropic resolution; 144 sagittal slices). Four whole‐brain anatomical images were acquired and further averaged for ROI localization and results display.

### EEG Data Analyses

The anesthesia level was evidently identified by EEG signals observed in the forehead,^[^ ^1^ ^]^ and EEG recordings only from the left frontal electrode were analyzed. First, fMRI gradient artifacts in the EEG were corrected using the fMRI artifact slice template removal method.^[^ ^67^ ^]^ Then, the DBSFILT toolbox (https://github.com/guillaumelio/DBSFILT) was applied to remove brief artifacts frequently induced by electrical stimulation. Next, preprocessing was conducted using the EEGLAB toolbox (https://sccn.ucsd.edu/eeglab/index.php). The data were downsampled to 1000 Hz and bandpass filtered (1–30 Hz). The cardio‐ballistic artifact was corrected via weighted average artifact subtraction.^[^ ^68^ ^]^ Spectrograms were computed using the multi‐taper method^[^ ^69^ ^]^ (window length = 4 s; step size = 0.05 s; spectral smoothing = ± 1 Hz; Slepian tapers = 7) in the Chronux toolbox (http://chronux.org). Beta (12–30 Hz) and delta (1–4 Hz) bands were extracted for computing the band power.

### fMRI Data Preprocessing

Preprocessing of fMRI images was performed using the statistical parametrical mapping toolbox (SPM8; http://www.fil.ion.ucl.ac.uk/spm), FMRIB Software Library toolbox (FSL; http://www.fmrib.ox.ac.uk) and Advanced Normalization Tools (ANTs; https://www.nitrc.org/projects/ants). Similar to what was done recently,^[^ ^70^ ^]^ after the first 10 volumes were discarded, DBS‐fMRI images were preprocessed using the following steps: slice timing and motion correction; linearly coregistered to the subject's high‐resolution anatomical image; spatially smoothed with a 4 mm Gaussian kernel. Note that extremely cautious steps were taken to minimize the influence of head motion. First, fMRI runs were excluded with the following thresholds: translation < 0.1 mm and rotation < 0.1° in any direction. Considering that arousal effects induced by stimuli might cause spontaneous head movements, a stimulus‐motion index was then developed to minimize the potential confounding effect. Based on the block design, a box‐car signal was defined with one indicating DBS‐on periods and zero indicating DBS‐off periods. Pearson's correlation coefficients were calculated between each of the six‐dimensional head motion derivatives and the box‐car signal, resulting in six stimulus‐motion indices for each fMRI run. fMRI runs were discarded with the following thresholds: maximum index < 0.35 and mean index < 0.15. Note that the entire run would be removed if any volume exceeded these extreme values. As such, 30 fMRI runs from four DBS‐fMRI sessions were included for statistical analysis (see Table S2, Supporting Information).

### Functional Profiles of the ILN‐DBS Driven Networks

Functional activation driven by ILN‐DBS was analyzed by applying a linear model with a block design (DBS‐on versus DBS‐off) in the SPM12 toolbox (Figure 2a). For each run, the 1st level activation map was estimated via the beta coefficient of the model. The resulting beta maps were subjected to a two‐level analysis (one‐sample *t*‐test). The significance level was set at voxel‐wise *p <* 10^−7^ and cluster‐level FWE corrected *p <* 0.05. The focus was on a consensus network consisting of ILN‐DBS‐driven regions in both subjects and a set of 24 regions was determined, including bilateral M1, PMCdl, PMCm, S1, S2, PCip, PCi, A2, TCc, TCs, VACd, and Put (Figure 3a), as a network robustly activated by ILN‐DBS. Considering the Tmax of the activation clusters, high‐resolution anatomical images, and the location of the electrodes, the mean time courses of these regions (radius = 2 mm, Figure 3a) were extracted^[^ ^71^ ^]^ to calculate stimulus‐locked changes in BOLD signals (BOLD% change) relative to DBS‐off epochs.

### Dynamics of the ILN‐DBS‐Driven Networks

The dynamic connectivity change of the ILN‐driven functional network was examined using the DynamicBC toolbox (https://www.nitrc.org/projects/dynamicbc). The preprocessed data were filtered (0.01–0.08 Hz) and regressed out the box‐car function convolved with the canonical HRF to remove its impact on FC estimation. A sliding window method with a window size of 10 TR and a window step of 1 TR was used to estimate FC dynamics. To assess the impact of stimulation at the node and network levels, graph metrics of each sliding window, were calculated such as node strength (NS, summation of FCs of each node) and connectivity strength (CS, summation of all FCs). Sliding windows containing > 50% stimulation period were classified as “DBS‐on”, whereas sliding windows containing = 0% stimulation period were classified as the “DBS‐off” condition. For dynamic FCs, cluster‐level correction of *p <* 0.05 was applied to adjust the multiple comparisons using the network‐based statistic,^[^ ^72^ ^]^ with the edgewise threshold of significance level set at *p <* 0.001. For dynamic node strength, the Bonferroni correction was considered for multiple comparisons, whereby the total number of adjusted tests was determined by the number of nodes. As a demonstration, the time course of dynamic graphmetrics was plotted, and a two‐sample t‐test was applied to assess group differences between conditions.

### Aligning CMBA Samples to the Arousal Network

Gene expression from the Cynomolgus Macaque Brain Atlas (CMBA) was analyzed, and a region‐specific RNA‐seq atlas was acquired from 878 tissue samples of the macaque brain. A complete description of the CMBA data can be found in the recent work^[^ ^29^ ^]^ and was briefly summarized here. After quality control, high‐quality reads were aligned to the cynomolgus macaque reference genome (Macaca_fascicularis_5.0) from the Ensembl database using STAR (v.2.7.3a)^[^ ^73^ ^]^ with command line “—runMode genomeGenerate” to build the sequence index. The entire mRNA mapping information was wrapped in the BAM format alignments. The corresponding gene annotation file for cynomolgus macaques was downloaded for reference alignment annotation and downstream quantification. PicardTools (v.2.21.2) was used to detect outlier samples after read alignment. The number of reads uniquely mapped to each gene was counted using featureCounts.^[^ ^74^ ^]^ Finally, the gene count matrix of 819 samples with 28 415 transcripts was retained.

### Network‐Based Differential Expression Analysis

Based on the organization of the ILN‐driven arousal network (Figure 3a), RNA‐seq samples were divided into two categories, within and outside the network, to identify a set of genes specifically expressed in the network compared to the rest of the brain. The previous gene count matrix in which 294 samples were assigned to the arousal network and 525 samples were assigned to the rest of the brain was used for input data. First, only genes expressed in ≥10% of samples were considered robustly expressed, and only protein‐coding genes were obtained using the corresponding gene transfer format file for Macaca fascicularis (v.5.0.102). Then, the Ensembl IDs were converted to gene symbols. Duplicate gene symbols were removed by retaining the maximum gene counts.

The limma package (v.3.52.4)^[^ ^75^ ^]^ in R was used to remove batch effects and perform differential expression gene (DEG) analysis. First, low‐expressed genes were filtered based on the max counts per million < 0.5 among samples. The design matrix was set up with both the grouping information and the batch information. The heteroscedasticity from RNA‐seq count data was removed using the voom method. Finally, linear models were used for comparisons and batch removal, and empirical Bayes moderation was carried out to obtain precise estimates of genewise variability. The reported *P* value was corrected for multiple tests using the Benjamini–Hochberg procedure to estimate the FDR. Genes with FDR corrected *p <* 10^−4^ were considered DEGs, shown as the volcano plot using the ggplot2 package (v.3.4.2).

### Gene Enrichment Analysis and PPI Network

The ClueGO^[^ ^76^ ^]^ plugin in Cytoscape (v.3.9.0)^[^ ^77^ ^]^ was applied to identify functionally enriched GO term networks of all the DEGs. Macaca mulatta was chosen as a reference organism for the annotation of the genes and selected GO terms of the BP, MF, and CC categories for gene enrichment analysis.^[^ ^78^ ^]^ Only GO terms with *p <* 10^−4^ (hypergeometric test, FDR corrected) were considered significant. Terms shared among a set of common genes were evaluated using the kappa statistic, and interrelatedness among nodes exceeding a kappa score of 0.5 was connected to each other.

GO pathway enrichment analyses for upregulated genes, downregulated genes, and overlapping genes were conducted separately using the clusterProfiler package (v.4.7.1)^[^ ^79^ ^]^ and the org.Mmu.eg.db database (v.3.15.0). Genes were mapped from the gene symbol to EntrezID, and enrichment analysis was performed using the “bitr” function and “enrichGO” function in the clusterProfiler package. The analysis calculates enrichment tests of GO terms based on hypergeometric distribution and subject to FDR correction. The top four statistically significant GO terms for each category were reported in the Results. Only terms with FDR‐corrected *p <* 0.05 were considered significantly enriched in this work.

Gene Set Enrichment Analysis (GSEA) was performed using the “gseGO” function in the clusterProfiler package and visualized using the GseaVis package in R. GO terms with |NES| > 1.4 and *p <* 0.01 (GSEA permutation test) were considered significant.

Protein‐protein interaction (PPI) network analysis was further applied to identify the submodules within the genes that belong to the “ion transport” GO term using stringApp in Cytoscape.^[^ ^77^ ^]^ Upregulated genes were imported to Cytoscape from the “STRING: protein query” data source to create the PPI network with a confidence cutoff of 0.7. The enrichment data were loaded using the “String Enrichment” option, and then the genes annotated to the “ion transport” GO term were selected to create a subnetwork. The largest subcluster of this network was plotted in the Results.

### Cell type‐Specific Gene Expression Analysis

To characterize whether these genes were overrepresented in specific cell types, single‐nucleus RNA‐sequencing (snRNA‐seq) data from Han et al. were used to identify distinct cell markers.^[^ ^30^ ^]^ This dataset consists of 11 cell types identified in the neocortex of Macaca fascicularis, including excitatory neurons, six subtypes of inhibitory neurons (IN; SST^+^, PVALB^+^, NPY^+^, LAMP5^+^, RELN^+^, VIP^+^), microglia, astrocytes, oligodendrocytes, and oligodendrocyte precursor cells. Briefly, the data were analyzed with published cell type annotations using the Seurat package (v.4.3.0)^[^ ^80^ ^]^ in R. Markers were identified between each cell type and all other cell types using the “FindMarkers” function based on the Wilcoxon‐rank sum test followed by FDR correction. For each cell type, markers were defined by *p <* 0.05 and log_2_(FoldChange) > 0.3.^[^ ^81^ ^]^ Gene set enrichment for each cell type was performed using Fisher's exact test with a threshold of FDR corrected *p <* 0.01.

For methodological validation, cell‐level enrichment was performed using the AUCell package (v.1.16.0),^[^ ^82^ ^]^ which utilizes the “area under the curve” (AUC) to determine whether the input gene set was enriched within the expressed genes for each cell. The AUC estimates the proportion of genes in the gene set that were highly expressed in each cell. The upregulated genes and downregulated genes were input for AUC score calculation. The “AUCell_calcAUC” function was used in the AUCell package to calculate the AUC score and kept all parameters as defaults. Then, thresholds were set using the function “AUCell_exploreThresholds” and manually adjusted to show cells with higher AUC scores. The thresholds were set as AUC > 0.14 for upregulated genes, AUC > 0.064 for downregulated genes, and AUC > 0.11 for overlapping upregulated genes. Then, cell clustering UMAP embedding was colored based on the AUC score of each cell to show which cell clusters were enriched in the upregulated genes and downregulated genes. Importantly, two additional snRNA‐seq datasets from Bo et al.^[^ ^29^ ^]^ and Ma et al.^[^ ^83^ ^]^ were further used for external validation.

### Layer‐specific Gene Expression Analysis

It was examined whether identified DEGs were particularly expressed in deeper cortical layers that preferentially possess cortico‐thalamic projection neurons. Cortical microarray datasets covering the dorsolateral prefrontal cortex and temporal area from Bernard et al. were used for analysis.^[^ ^31^ ^]^ Of those genes that were differentially expressed in the arousal network, 1764 genes were identifiable in the microarray data from Bernard et al. The limma package was used to detect differentially expressed genes between cortical layers 2/3 and 5/6 among the DEGs in the arousal network. Genes with *p <* 0.05 were considered layer‐specific expressed genes. The expression values of these layer‐specific expressed genes were then hierarchically clustered using an Euclidean distance dissimilarity function and plotted using the gplots package in R.

### Gene Expression Analysis in Humans

The Abagen toolbox^[^ ^84^ ^]^ was used to process transcriptomic data from the Allen Human Brain Atlas (AHBA)^[^ ^32^ ^]^ and mapped the transcriptomic data onto the identified arousal network in a similar manner. First, microarray probes were reannotated,^[^ ^85^ ^]^ and probes without matching to a valid Entrez ID were discarded. Second, probes with intensities less than the background in > = 50% of samples across donors were discarded,^[^ ^86^ ^]^ resulting in 31 569 probes. When multiple probes indexed the expression of the same gene, the probes were kept with the highest mean intensity across samples. The selection of probes was performed using sample expression data aggregated across all donors, yielding 15 633 genes. Third, samples from each donor were assigned inside or outside the arousal network according to their MNI coordinates and averaged for each donor.

Using the limma package, gene‐wise linear modeling was performed to identify DEGs by comparing the given gene expression within and outside the arousal network. Residual donor effects were eliminated using Limma's “duplicateCorrelation” function.^[^ ^9^ ^]^ Genes with FDR‐corrected *p <* 10^−4^ were considered DEGs.

### Statistical Analysis

All statistical analyses except bioinformatics analyses were conducted in MATLAB (R2017a, MathWorks, USA) utilizing SPM12 and other toolboxes mentioned above. Bioinformatic analyses were conducted using R (v.4.2.3, https://www.r‐project.org/) utilizing specific R packages noted above. Statistical significance was calculated as noted in the corresponding Figure Legends and Results sections.

## Conflict of Interest

The authors declare no conflict of interest.

## Author Contributions

Z.Z. and Y.C.H. contributed equally to this work. Z.W., Y.W., and M.Y.W. contributed to the conception and design of the study. Z.Z., Y.C.H., X.Y.C., J.H.L, Y.Y., L.B.L., J.H.W., M.Y.W., Y.W., and Z.W. contributed to the acquisition, post‐processing, and analysis of the data. Z.Z., X.Y.C., and Y.C.H. analyzed all or parts of the data, drafted the text, and prepared the figures with the help of Z.W. Z.W. wrote the manuscript, obtained the findings, and supervised the study. All authors approved the final version of the manuscript.

## Supporting information

Supporting Information

## Data Availability

The data that support the findings of this study are available from the corresponding author upon reasonable request.
