# Supplementary material for: State‐specific Regulation of Electrical Stimulation in the Intralaminar Thalamus of Macaque Monkeys: Network and Transcriptional Insights into Arousal
Source: Adv Sci (Weinh). 2024 Jun 27;11(33):2402718. doi: 10.1002/advs.202402718 (PMC11434125; doi:10.1002/advs.202402718)
Supplement: Supplementary file 1 — Supporting Information [file ADVS-11-2402718-s001.pdf]

ADVANCED SCIENCE
Open Access

Supporting Information

for *Adv. Sci.*, DOI 10.1002/advs.202402718

State-specific Regulation of Electrical Stimulation in the Intralaminar Thalamus of Macaque Monkeys: Network and Transcriptional Insights into Arousal

Zhao Zhang, Yichun Huang, Xiaoyu Chen, Jiahui Li, Yi Yang, Longbao Lv, Jianhong Wang, Meiyun Wang, Yingwei Wang* and Zheng Wang**

Supporting Information

State-specific Regulation of Electrical Stimulation in the Intralaminar Thalamus of Macaque Monkeys: Network and Transcriptional Insights into Arousal

Zhao Zhang, Yichun Huang, Xiaoyu Chen, Jiahui Li, Yi Yang, Longbao Lv, Jianhong Wang, Meiyun Wang, Yingwei Wang*, Zheng Wang**

*Correspondence: Zheng Wang, PhD; Yingwei Wang, MD, PhD; Meiyun Wang, MD, PhD

E-mail: zheng.wang@pku.edu.cn; wangyw@fudan.edu.cn; mywang@ha.edu.cn

This file includes:

Supporting text

Figure S1 to S6

Table S1 to S4

SI References

Supporting Information Text

Below are the details directly referenced in the main document.

A. Additional Results

FC profiles of ILN at finely-tuned propofol anesthesia levels. We first collected a series of resting-state fMRI (RS-fMRI) data sessions, and investigated the functional connectivity profile of ILN (ILN-FCs) in monkeys at distinct propofol-induced anesthetized states (EEG slow-wave oscillations (SW) and isoelectric lines (IsoE)). An example session of propofol infusion protocol and EEG-fMRI data acquisition showed pronounced changes in amplitude and power of EEG signals (sampled from left frontal electrode) during different epochs in Figure S1a. The SW epochs before and after IsoE were defined as the baseline and recovery SW (b-SW and r-SW), respectively. The unprocessed EEG recordings clearly exhibited a substantial reduction in amplitude during IsoE epoch relative to SW epochs, which was also demonstrated by the corresponding spectrogram shown in the below. Statistical comparison of mean blood pressure, end-tidal carbon dioxide and body temperature of subjects showed no significant differences among three epochs, although the heart rate decreased significantly during IsoE epochs (Figure S1b, repeated measures ANOVA; ***$P < 0.001$, *$P < 0.05$, Tukey-Kramer multiple comparison test).

The ILN including the centromedian-parafasicularis complex (CMn-PF) and the central lateral and paracentralis nuclei (CL-PC) were identified from the SARM atlas (Figure S2a) and used as seeds to calculate the voxel-wise functional connectivity map. Group-level ILN-FCs computed with one-sample t-test in each epoch are presented in Figure S2b (left panel; voxel-wise $P < 0.01$, FDR corrected, cluster ≥ 8 voxels). In b-SW epoch, the BOLD activity in ILN was positively correlated with cortical regions (Table S1) including the primary motor cortex (M1), medial premotor cortex (PMCm), primary somatosensory cortex (S1), intraparietal cortex (PCip), centrolateral prefrontal cortex (PFCcl), primary visual cortex (V1) and posterior cingulate cortex (PCC). These connectivities were significantly suppressed in IsoE epoch, and partially recovered in r-SW epoch. To quantitatively compare the connectivity strength of ILN-FCs among

three epochs, we identified seven cortical clusters from ILN-FCs in b-SW epoch and calculated the mean connectivity across all cortical clusters or specific thalamus (SFT). Results showed a remarkable reduction in ILN-cortical connectivity in IsoE epoch that partially recovered in r-SW epoch, whereas only a mild reduction in ILN-SFT connectivity was found in IsoE epoch (Figure S2b, right panel; *** $P < 0.001$, ** $P < 0.01$, * $P < 0.05$, Tukey-Kramer multiple comparison test).

We further conducted connectivity analysis of two subareas of ILN (i.e., CL-PC nuclei and CMn-PF nuclei, as highlighted in Figure S2a) with seven cortical regions between b-SW epoch and IsoE epoch. Group differences are represented using effect size (Hedges' g value, bottom-left triangle in Figure S2c, left panel) and corresponding *P* value ($P < 0.05$, NBS correction with edge-wise $P < 0.001$; top-right triangle in Figure S2d, left panel). Evidently, connectivity between areas including PCip, PMCm, PCC, M1, and S1 were significantly decreased, among which PMCm had the largest effect size. Within the ILN, the connectivity network of CMn-PF nuclei appeared to be largely affected by high-dose propofol relative to the CL-PC nuclei (Figure S2c, right panel; * $10^{-4} < P < 0.01$, ** $P < 10^{-4}$).

B. Supplementary Methods for section A

EEG-fMRI data acquisition

The procedure of simultaneous EEG-fMRI data acquisition was similar to Materials and Methods. After preparation, monkeys were transferred to the scanner room and ventilated with 50% oxygen via an MRI-compatible ventilator (CWE Inc., Weston, Wisconsin) by intermittent positive-pressure ventilation to ensure a constant breath rate (25-35 breaths/min). EEG signals from nineteen active electrodes were sampled at 5000 Hz with a resolution of 0.5 mV per bit and a range of ±16 mV. The recording clocks of MRI console and EEG recording system were synchronized using the SyncBox for offline artifact removal in EEG signals.

RS-fMRI was applied during different anesthesia levels defined by real-time EEG recordings. Figure S1a displays the timeline of RS-fMRI experiment and a total of 8

fMRI sessions were acquired from 8 participants (Table S2). At the beginning of each session, the infusion rate of propofol (15.87 ± 4.17 mg/kg/h) was adjusted based on real-time EEG and physiological monitoring to reach a stable state with EEG slow-wave oscillations (defined as b-SW epoch). Then, the infusion rate was gradually increased to 61.5 ± 17.61 mg/kg/h to reach a stable state with continuous isoelectric-lines in EEG (IsoE epoch). Note that phenylephrine was applied to maintain mean blood pressure close to the b-SW state in this epoch. Lastly, the infusion was suspended until no suppression was detected in EEG for at least five minutes, and the infusion was resumed (12 ± 1.51 mg/kg/h) to maintain a stable state with EEG slow-wave oscillations (r-SW epoch). We followed Barttfeld's steps[1] to apply the muscle relaxant (cisatracrium, 0.15 mg/kg bolus i.v., followed by continuous i.v. infusion at a rate of 0.18 mg/kg/h; Jiangsu Hengrui Pharma Co., Ltd.). In brief, each RS-fMRI session contained three epochs: b-SW epoch (4 RS-fMRI runs), IsoE epoch (8 RS-fMRI runs), and r-SW epoch (4 RS-fMRI runs).

RS-fMRI data were collected using a custom-made bird-cage volume coil with 8-channel phase-array receiver coils. Functional images were acquired using a gradient-echo EPI sequence (TR = 2000ms; TE = 29 ms; flip angle = 77°; slices = 32; matrix = 64 × 64; FOV = 96 mm × 96 mm; 1.5 mm × 1.5 mm in plane resolution; slice thickness = 2.5 mm) for a total of 200 functional volumes. A pair of gradient-echo images (echo time: 4.22 and 6.68 ms) with the same orientation and resolution as the EPI images were acquired to generate a field map for distortion correction of EPI images. T1-weighted anatomical images were acquired using a MPRAGE sequence (TR = 2300 ms; TE = 3.12 ms; TI = 1000 ms; flip angle = 9°; 0.5mm isotropic resolution; slices = 144). Six whole-brain anatomical runs were collected and averaged for better brain segmentation and registration.

EEG data analyses

EEG recordings from the left frontal electrode were preprocessed as follows: 1) fMRI gradient artifacts in the EEG were corrected using the fMRI artifact slice template removal method; 2) The data were down-sampled to 1000 Hz and band-pass filtered

(1-30 Hz); 3) The cardio-ballistic artifact was corrected via weighted average artifact subtraction. Spectrograms were computed using the multi-taper method (window length = 4 s; step size = 0.05 s; spectral smoothing = ± 1 Hz; Slepian tapers = 7).

fMRI data analyses

fMRI data preprocessing After the first 10 volumes were discarded, fMRI images were preprocessed using the following steps: Firstly, we applied the field map images of each session to compensate for the geometric distortion of EPI images caused by magnetic field inhomogeneity using FSL FUGUE. After slice timing and motion correction, the corrected images were co-registered (six degrees of freedom rigid transformation) to the subject's anatomical image and normalized (optimum 12-parameter affine transformation and nonlinear deformations) to F99 template (http://sumsdb.wustl.edu/sums/macaquemore.do). All functional images were resampled to 2 mm cubic voxels, and spatially smoothed with a 4 mm full-width at half-maximum (FWHM) isotropic Gaussian kernel. Further denoising steps included linear detrending, regressing out nuisance covariates (six head motion parameters, white matter and ventricle signals), and temporal filtering (0.01–0.08 Hz).

Functional connectivity analyses Based on the co-registered T1 images, the SARM atlas was used to segment the ILN, which was further divided into anterior section (mainly the central lateral and paracentral nuclei, CL-PC) and posterior section (mainly the centromedian-parafasicularis complex, CMn-PF). The remaining part of thalamus was defined as specific thalamus (SFT). Hence bilateral ILN were used as seeds to obtain averaged time course of fMRI signals and then treated as a regressor of interest in a linear regression model to estimate its functional connectivity strength with the rest of brain (i.e., beta coefficients). The voxel-based connectivity strength was statistically tested in each epoch using one-sample *t*-test (voxel-wise $P < 0.01$ and cluster-level FDR corrected $P < 0.05$). Based on Regional Map atlas, those statistically significant clusters within cortex or SFT were extracted (Table S1) to compare between states using repeated measures ANOVA: one fixed factor was set and examined, i.e., anesthesia level (IsoE versus SW epochs).

The functional connectivity analysis was conducted between these cortical clusters and ILN subareas using Pearson's correlation coefficients. The correlation coefficients were then normalized to *z*-scores using Fisher's transformation. Group difference between b-SW and IsoE epochs was evaluated using two-sample *t*-test. Edgewise threshold of the significance level was set at $P < 0.001$, and the corresponding effect size was measured by *Hedges' g* value. Cluster-level correction of $P < 0.05$ was applied to adjust the multiple comparison using the network-based statistic.

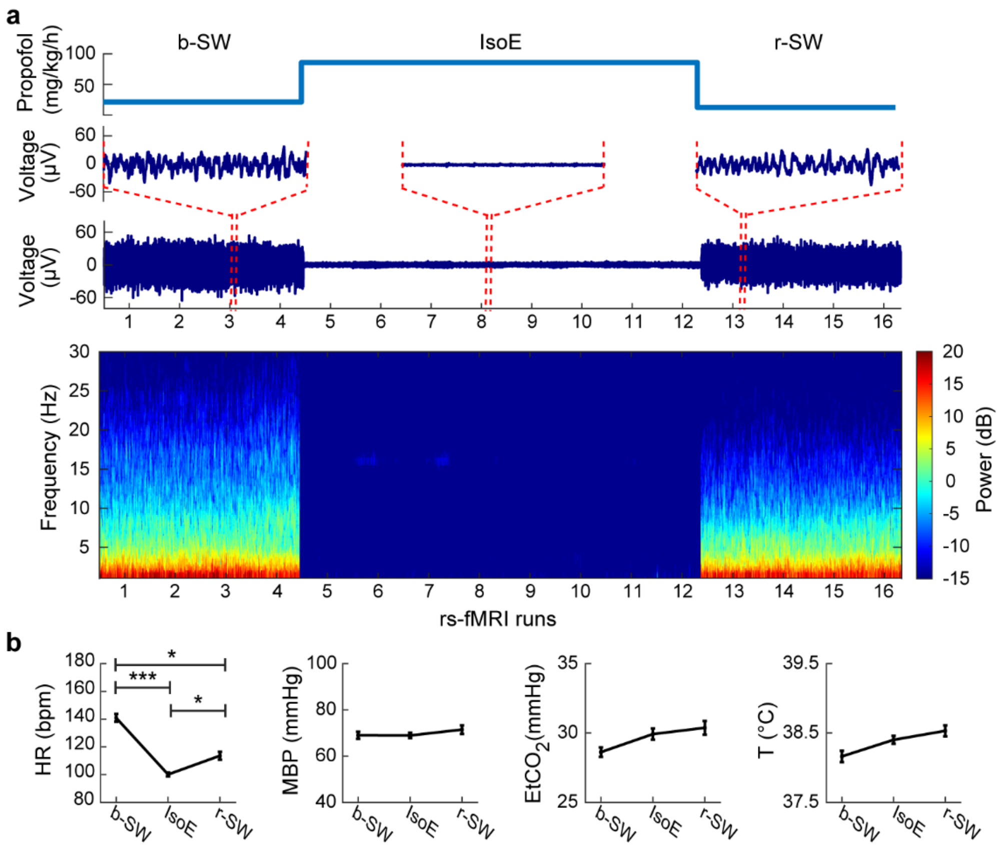

Figure S1. Resting-state experimental design and physiological responses to graded anesthesia levels. a) Example trace of propofol infusion rate, unprocessed EEG signals and spectrogram simultaneously recorded from the left frontal electrode during resting-state fMRI runs when EEG showed stable slow-wave oscillations (b-SW epoch, 4 runs), continuous isoelectric-lines (IsoE epoch, 8 runs), and restored slow-wave oscillations (r-SW epoch, 4 runs). Each run was 400s. The magnified view displays 20s EEG signals from each epoch. b) Vital signs across three epochs were compared by repeated measures ANOVA. Data are shown as mean ± SEM. * $P < 0.05$, *** $P < 0.001$, Tukey-Kramer multiple comparison test. HR, heart rate; MBP, mean blood pressure; $EtCO_2$, end-tidal carbon dioxide; T, temperature.

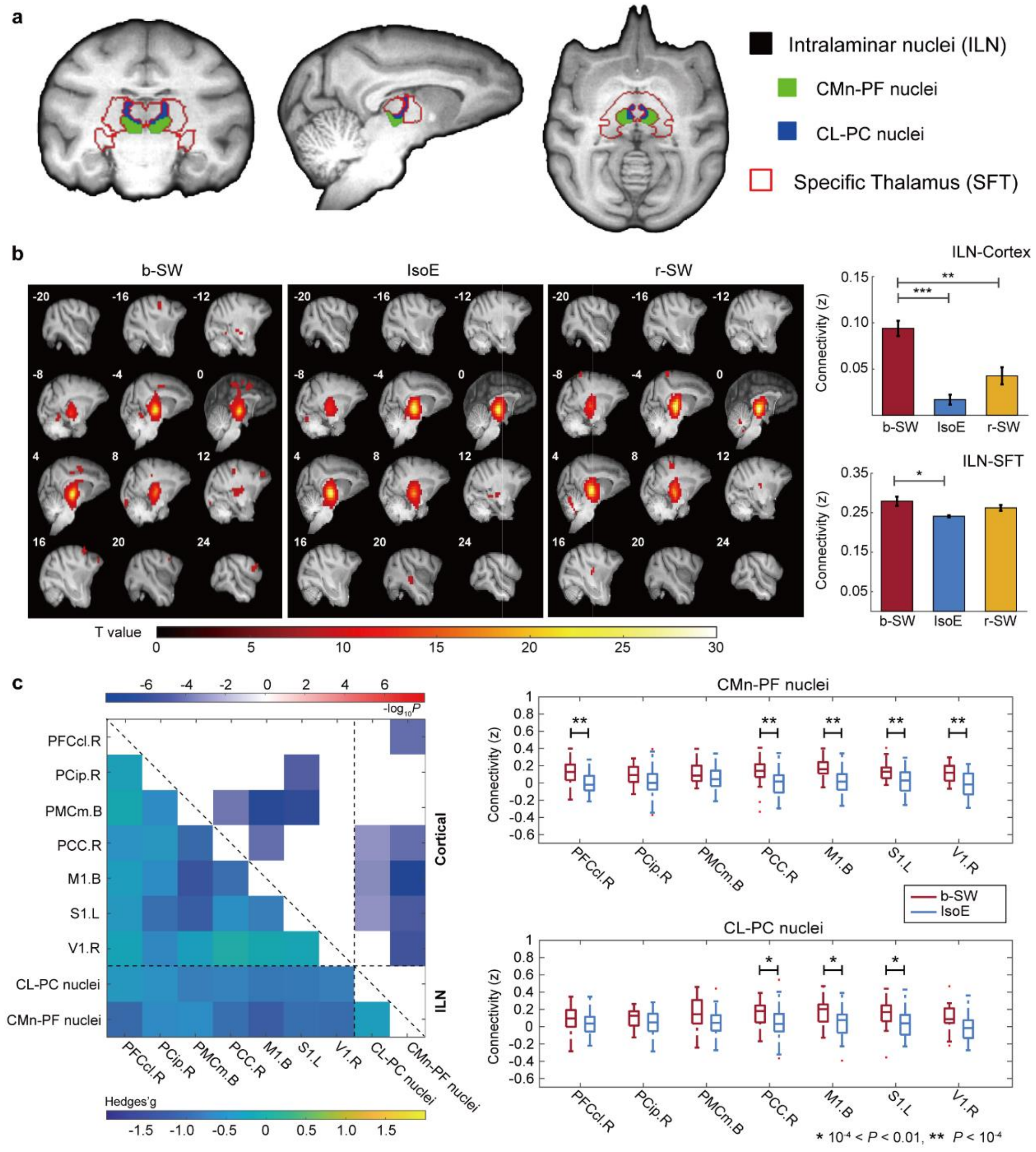

Figure S2. FC profiles of ILN at finely-tuned propofol anesthesia levels. a) Subareas of thalamus from Subcortical Atlas of the Rhesus Macaque (SARM). Coronal, sagittal, and axial slices show the same atlas overlaid on a standard F99 template. ILN was highlighted in solid, which can be sub-divided into CMn-PF nuclei (solid green) and CL-PC nuclei (solid blue). The rest of thalamus was defined as SFT which was highlighted by red lines. CL-PC, centrolateral-paracentral; CMn-PF, centromedial-parafascicular; ILN, intralaminar nuclei; SFT, specific thalamus. b) Group level voxel-wise connectivity map of bilateral ILN (ILN-FCs) in b-SW, IsoE, and r-SW epochs

(one sample t test; $P < 0.01$, FDR corrected). In IsoE epoch, connectivity strength between ILN and cortex was significantly suppressed (right upper panel), whereas between ILN and SFT was only mildly inhibited (right lower panel; * $P < 0.05$, ** $P < 0.01$, *** $P < 0.001$, Tukey-Kramer multiple comparison test). Data are shown as mean ± SEM. SW, slow-wave. c) Altered functional connections within ILN-FCs during the IsoE epoch compared to the b-SW epoch. Left panel shows the altered functional connectivity: effect sizes (*Hedges' g* value) in lower triangle and p-values ($P < 0.05$, NBS correction with edge-wise $P < 0.001$) in upper triangle. In right panel, the box-plot shows the altered functional connectivity between CMn-Pf / CL-PL nuclei and cortical clusters. Centre bar shows median, bottom and top of box designate 25th and 75th percentiles respectively, and the solid red points indicate data extremes. *$10^{-4} < P < 0.01$, ** $P < 10^{-4}$.

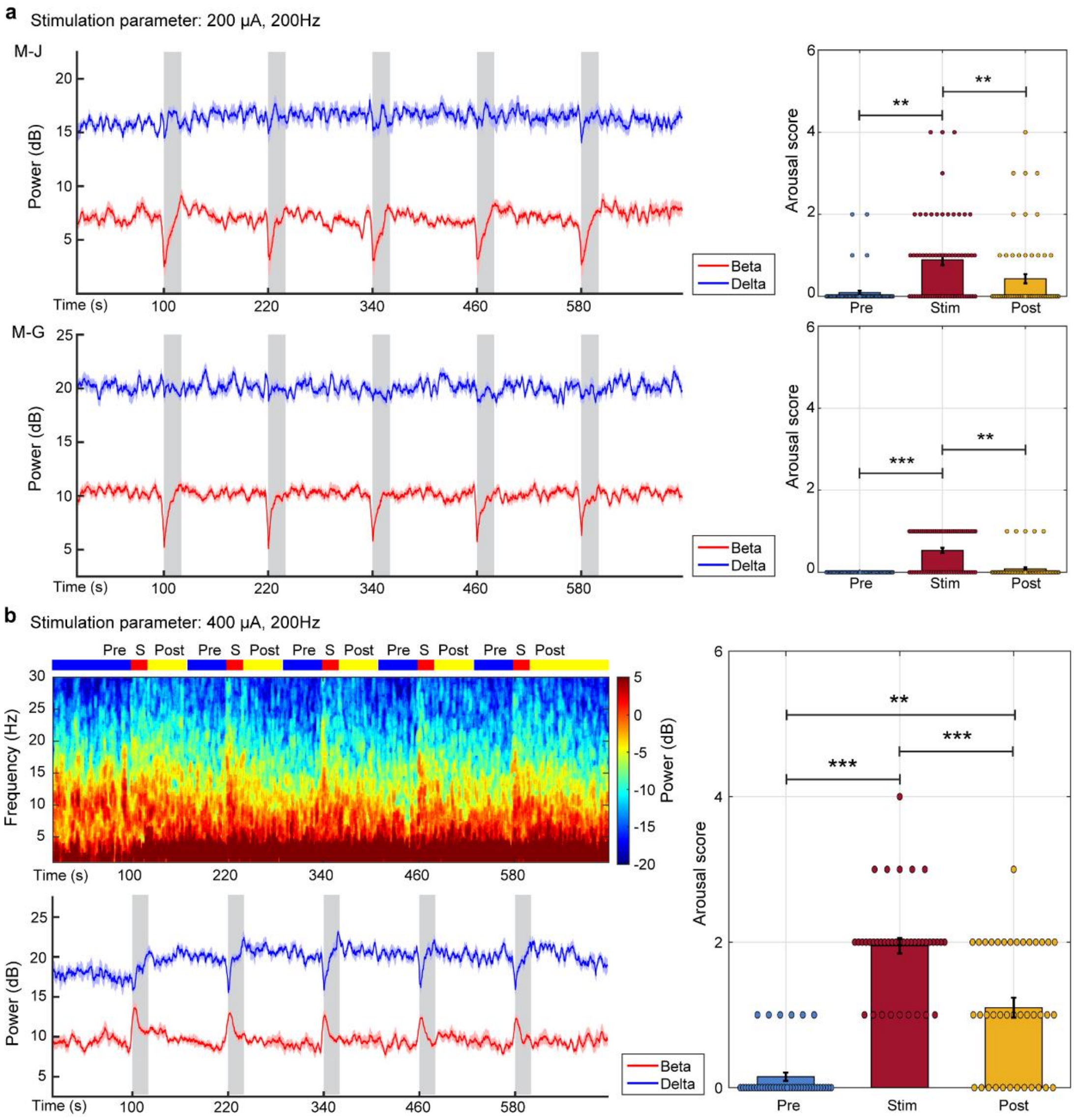

Figure S3. Physiological and behavioral effects of ILN-DBS during propofol induced slow-wave oscillations. a) Trend of group mean band power of M-J with 200-μA stimulation (upper left panel). Solid line indicates mean power averaged across stimulation runs, whereas the shaded areas represent ±SE. Group mean arousal score (±SE) before, during, and after stimulations of the same monkey (upper right panel, n = 65). Circles show individual stimulation events. Group mean band power (lower left panel) and arousal score (lower right panel) of M-G (n = 60). b) Example EEG spectrogram of left frontal electrode in an example run (upper left panel) and trend of population mean band power (lower left panel) with 400-μA stimulation. Population

mean arousal score (±SD) before, during, and after stimulations for both monkeys ($n = 40$). ** $P < 0.01$, *** $P < 0.001$, Tukey-Kramer multiple comparison.

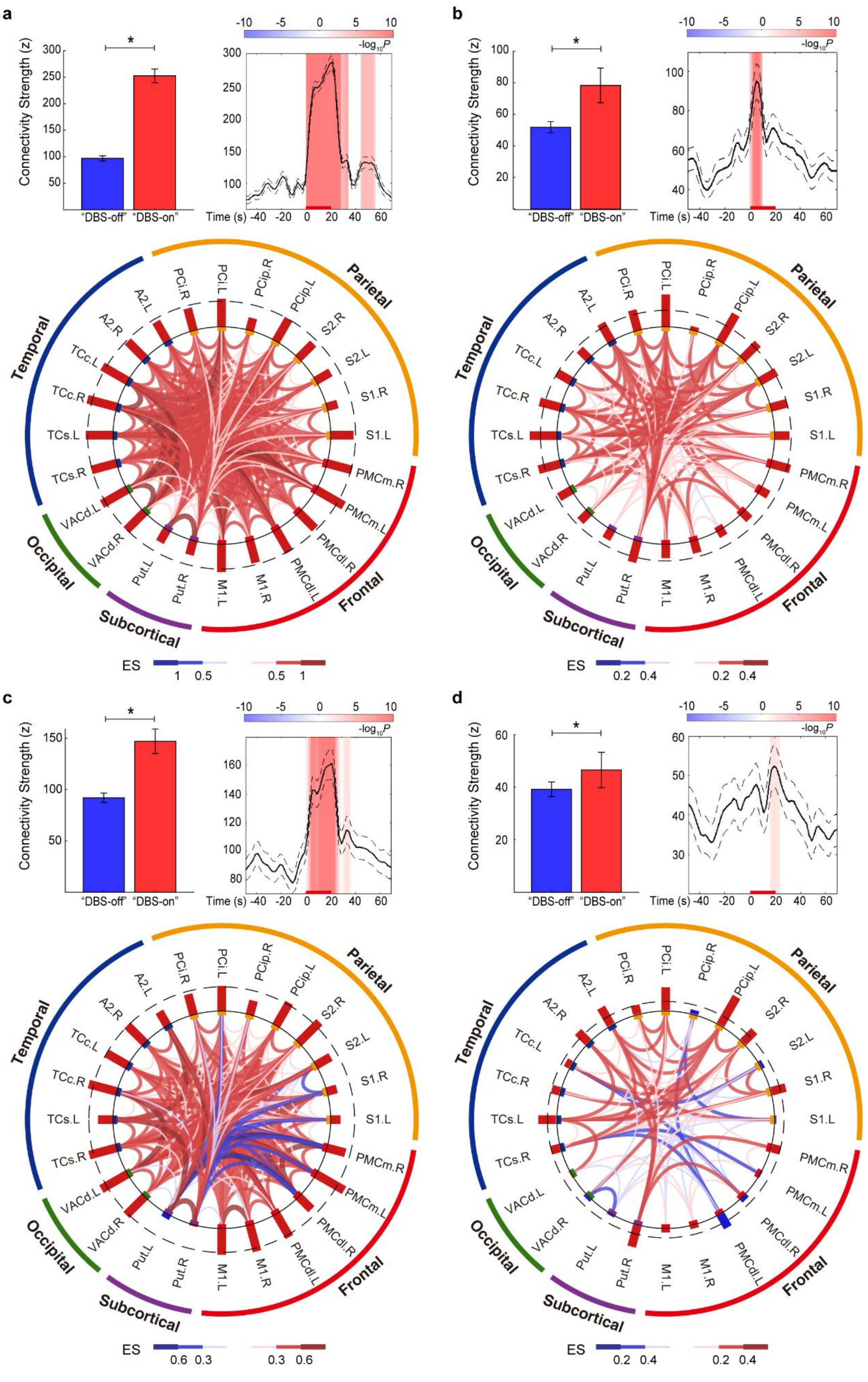
a
b
c
d
Connectivity Strength (z)
"DBS-off"
"DBS-on"
-log10P
Time (s)
Temporal
Parietal
Occipital
Subcortical
Frontal
PCi.L
PCi.R
PCip.R
PCip.L
A2.L
A2.R
S2.R
S2.L
S1.R
S1.L
TCc.L
TCc.R
TCs.L
TCs.R
PMCm.R
PMCm.L
VACd.L
VACd.R
PMCdl.R
PMCdl.L
Put.L
Put.R
M1.L
M1.R
ES

Figure S4. Dynamic functional connectivity analysis of datasets with varying motion screening thresholds or regression covariates. Datasets with motion screening thresholds of maximum SMI < 0.4 and mean SMIs < 0.3 from M-J a) and M-G b). Connectivity strength (CS) shows significant enhancement during "DBS-on" condition ($P < 0.001$; upper left panel). The time course of CS is also plotted according to the stimulation block with solid and dashed lines as mean ± SEM (upper right panel). Stimulation onset is aligned to 0 s and stimulation period is marked in red from 0 to 20 s (10 TRs). Time points with significantly increased or decreased ($P < 0.001$; two-sample t-test) CS than that in "DBS-off" condition are highlighted in red or blue, respectively. Color bar indicates the p-values. Altered functional connections with node information are plotted in lower panel. Red or blue lines respectively label significantly increased or decreased connections ($P < 0.05$, NBS correction with edge-wise $P < 0.001$), with width and color indicating effect size (*Hedges' g* value). For nodes, red and blue bars in the interlayer indicate the effect sizes (*Hedges' g* value) of increased and decreased node strength, respectively. The dashed line labels the top 50% nodes with significantly altered node strength. Datasets with regressing out covariates including six head motion parameters, white matter and ventricular signal from M-J c) and M-G d). Statistical significance for CS was set at p=0.05. A2, secondary auditory cortex; M1, primary motor cortex; PCi, inferior parietal cortex; PCip, intraparietal cortex; PMCdl, dorsolateral premotor cortex; PMCm, medial premotor cortex; Put, putamen; S1, primary somatosensory cortex; S2, secondary somatosensory cortex; TCc, central temporal cortex; TCs, superior temporal cortex; VACd, anterior visual area (dorsal part).

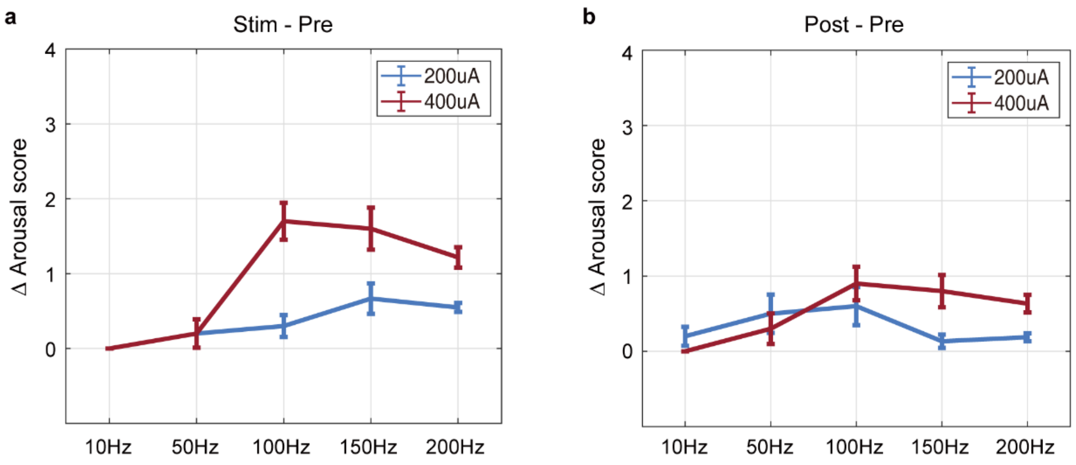

Figure S5. Arousal effect of ILN-DBS using different stimulation parameters. Changes in arousal score under stimulation versus pre-stimulation baseline a) and post-stimulation versus pre-stimulation baseline b). Blue and red lines respectively indicate the change of arousal score from 200 μA and 400 μA-stimulation at different frequencies. Data are shown as mean ± SEM. The results show an obvious stimulation-induced arousal using parameters of 200 μA and 200 Hz, with less arousal effect in post-stimulation period.

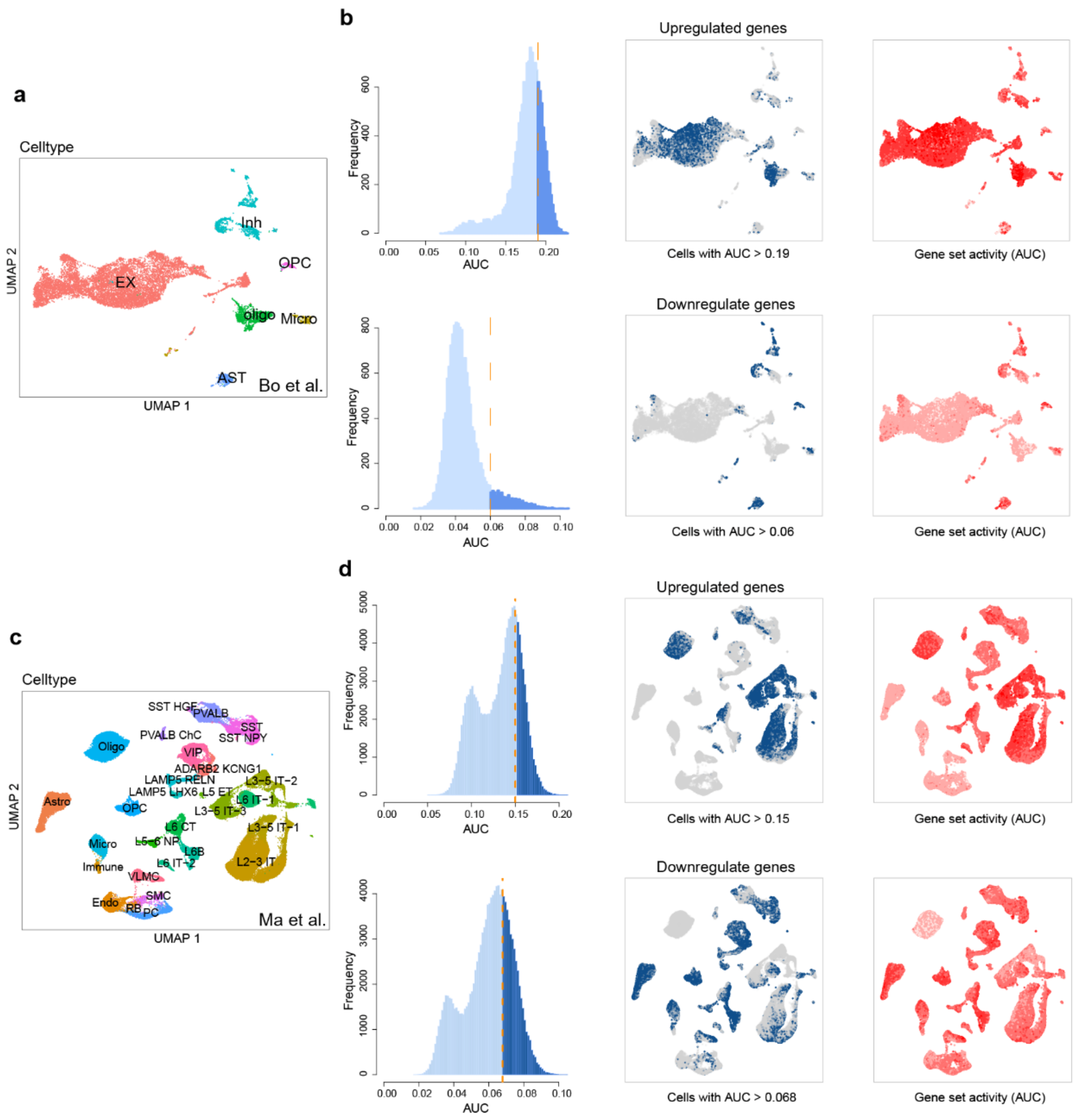

Figure S6. Cell cluster annotations and cell type enrichment of DEGs annotated with two independent snRNA-seq datasets. a) UMAP visualization of cell type clusters from Bo et al. b) Cell-level enrichment of upregulated genes (upper panel) and downregulated genes (lower panel) using AUCell with the data from Bo et al. The AUC threshold was set as 0.19 and 0.06 for upregulated genes and downregulated genes, respectively. c) UMAP visualization of cell type clusters from Ma et al. d) Cell-level enrichment of upregulated genes (upper panel) and downregulated genes (lower panel) using AUCell with the data from Ma et al. The AUC threshold was set as 0.15 and 0.068 for upregulated genes and downregulated genes, respectively. UMAP, Uniform Manifold Approximation and Projection; EX, excitatory neurons; IN, inhibitory

neurons; OPC, oligodendrocyte precursor cells; AST, astrocyte; IT, intratelencephalic; ET, extratelencephalic; NP, near-projecting; CT, corticothalamic; ChC, chandelier cells; Endo, endothelial cells; RB, red blood lineage cells; PC, pericyte; SMC, smooth muscle cells; VLMC, vascular leptomeningeal cells.

Table S1. ILN-FCs in the base-SW epoch*

	Location	Abbr.	Coordinates (x y z)			Cluster size (mm^3)	Max T-value
Cortical	Primary motor cortex (B)	M1	-17	-6	16	224	5.31
	Medial premotor cortex (B)	PMCm	3	-4	18	176	5.13
	Primary somatosensory cortex (L)	S1	0	-16	20	88	4.92
	Intraparietal cortex (R)	PCip	13	-18	18	80	5.25
	Centrolateral prefrontal cortex (R)	PFCcl	11	16	16	72	4.73
	Primary visual cortex (R)	V1	9	-46	-6	64	5.33
	Posterior cingulate cortex (R)	PCC	1	-12	16	64	4.63
Sub-cortical	Specific thalamus (B)	SFT	-3	-12	0	2072	48.35

Note: Coordinates in standard F99 space (mm): x = mediolateral, y = rostrocaudal, and z = dorsoventral.

B, Bilateral; R, Right; L, left.

*voxel-wise ROI analysis (P < 0.01, FDR corrected; cluster ≥ 8 voxels).

Table S2. Summary of datasets

Datasets		Sessions	Runs collected	Runs used
RS-fMRI experiment (N=8)		8	128	128
DBS-behavioral experiment (N=2)	SW state	4	25 (200 µA)<br>8 (400 µA)	25 (200 µA)<br>8 (400 µA)
	IsoE state	2	10 (200 µA)<br>10 (400 µA)	10 (200 µA)<br>10 (400 µA)
DBS-fMRI experiment (N=2)		4	46 (200 µA)	30 (200 µA)[†]<br>37 (200 µA)[‡]

RS-fMRI, resting-state fMRI; SW, slow wave; IsoE, isoelectric-lines.

[†] runs with motion screening thresholds of maximum SMI < 0.35 and mean SMIs < 0.15

[‡] runs with motion screening thresholds of maximum SMI < 0.4 and mean SMIs < 0.3

Table S3. Parcellation and abbreviation from the Regional Map Atlas

Lobes	Abbreviation	Full name
Occipital	V1	Visual area 1 (primary visual cortex)
	V2	Visual area 2 (secondary visual cortex)
	VACv	Anterior visual area, ventral part
	VACd	Anterior visual area, dorsal part
Parietal	S1	Primary somatosensory cortex
	S2	Secondary somatosensory cortex
	PCm	Medial parietal cortex
	PCip	Intraparietal cortex
	PCi	Inferior parietal cortex
	PCs	Superior parietal cortex
Temporal	A1	Primary auditory cortex
	A2	Secondary auditory cortex
	TCpol	Temporal polar cortex
	TCi	Inferior temporal cortex
	TCv	Ventral temporal cortex
	TCc	Central temporal cortex
	TCs	Superior temporal cortex
	HC	Hippocampus
	PHC	Parahippocampal cortex
	Amyg	Amygdala
Frontal	M1	Primary motor cortex
	PMCvl	Ventrolateral premotor cortex
	PMCdl	Dorsolateral premotor cortex
	PMCm	Medial premotor cortex
	FEF	Frontal eye field
	PFCvl	Ventrolateral prefrontal cortex
	PFCcl	Centrolateral prefrontal cortex
	PFCdl	Dorsolateral prefrontal cortex
	PFCdm	Dorsomedial prefrontal cortex
	PFCm	Medial prefrontal cortex
	PFCpol	Prefrontal polar cortex
	PFCoi	Orbitoinferior prefrontal cortex
	PFCom	Orbitomedial prefrontal cortex
	PFCol	Orbitolateral prefrontal cortex
Cingulate	CCs	Subgenual cingulate cortex
	CCp	Posterior cingulate cortex
	CCr	Retrosplenial cingulate cortex

	CCa	Anterior cingulate cortex
Insula	G	Gustatory cortex
	Ia	Anterior insula
	Ip	Posterior insula
Subcortical	Cau	Caudate
	Put	Putamen
	Tha	Thalamus
	HT	Hypothalamus
	Nacc	Nucleus accumbens
	GP	Globus pallidus

Table S4. Detailed information for interlayer differential genes between layer 2/3 (upper layers) and layer 5/6 (deeper layers).

Gene name	log_2FoldChange	FDR corrected *P* value	Gene name	log_2FoldChange	FDR corrected *P* value
SLC12A5	0.35608	4.48×10^{-4}	GADD45A	-0.65191	3.08×10^{-9}
ADARB1	0.370662	0.004156	GLCCI1	-0.70611	3.08×10^{-9}
ARIH2	0.413094	0.002609	CCDC69	-0.43736	0.039856
CHRNA7	0.458329	1.02×10^{-5}	TMEM155	-0.54222	7.40×10^{-4}
RBFOX1	0.302914	0.006107	CAMK2D	-0.67834	6.15×10^{-6}
HIVEP2	0.377737	2.99×10^{-5}	ST8SIA1	-0.57485	0.001142
SPIRE1	0.189675	0.044254	NCK2	-0.37722	2.12×10^{-4}
SLC1A2	0.26544	0.005923	UGP2	-0.322	0.048515
ROCK2	0.501444	5.95×10^{-5}	ZNF385B	-0.59191	1.16×10^{-4}
COG2	0.328588	0.019536	LTB	-0.72536	9.11×10^{-5}
LZTS3	0.488336	7.17×10^{-9}	NT5DC1	-0.43067	0.015953
LVRN	0.354552	5.10×10^{-6}	HSD11B1	-0.77368	1.08×10^{-4}
UBASH3B	0.512326	6.30×10^{-9}	FAP	-0.74579	8.98×10^{-8}
SLC4A3	0.407988	2.86×10^{-5}	ASAP2	-0.95294	2.22×10^{-11}
KLHDC8B	0.440361	2.08×10^{-6}	CSRP2	-0.68769	8.95×10^{-6}
ITGAM	0.35095	0.001011	SULF2	-0.9618	3.02×10^{-11}
SRXN1	0.430645	1.74×10^{-5}	EFHD2	-0.83553	2.54×10^{-8}
LRRC4	0.456892	1.18×10^{-6}	ADGRG6	-0.53914	0.038509
SNN	0.55732	1.08×10^{-5}	TM6SF1	-0.55331	4.90×10^{-4}
KCNS2	0.526651	9.86×10^{-6}	BEND5	-0.39591	0.002258
CAMKK2	0.471063	0.002427	DRC1	-0.81745	4.08×10^{-5}
KCNC1	0.459921	3.84×10^{-4}	TIGAR	-0.65776	0.016066
THTPA	0.281784	0.040041	POPDC3	-1.3681	1.40×10^{-10}
SLC38A11	0.542202	1.32×10^{-4}	UST	-0.79375	1.79×10^{-6}
OSBPL2	0.243928	0.029893	PLCL1	-0.77927	1.11×10^{-7}
ABT1	0.254032	0.049235	ST3GAL6	-0.96594	1.28×10^{-5}
DNAJA4	0.319742	0.001079	AQP3	-1.2141	2.61×10^{-5}
GLS2	0.198023	0.018759	AQP1	-1.54691	2.49×10^{-7}
RNF115	0.293393	0.003348	CNIH3	-1.96333	2.81×10^{-14}
FHL2	0.320854	0.004158	RSPO3	-2.21213	7.50×10^{-13}

CKAP2	0.227025	0.031423	PLP1	-2.1695	1.41×10^{-12}
BRINP1	0.176162	0.022315	MBP	-2.35663	1.08×10^{-12}
SAT2	0.22018	0.035177	GPR37	-2.23747	1.24×10^{-12}
FNDC4	0.248213	0.00463	BCAS1	-2.13061	1.41×10^{-12}
MRPS7	0.280085	0.042651	KLK6	-2.11888	7.50×10^{-13}
SPECC1	0.408822	3.34×10^{-5}	ANLN	-2.11556	4.89×10^{-13}
MTCL1	0.332284	9.51×10^{-4}	SPP1	-1.82495	1.02×10^{-11}
ATP1A1	0.269308	0.004564	UGT8	-2.23455	1.44×10^{-11}
OSBPL1A	0.244288	0.004971	PLLP	-2.03415	4.03×10^{-11}
ZADH2	0.22452	0.043616	CARNS1	-1.90366	1.63×10^{-12}
PARD6A	0.289238	0.00312	FAM107B	-1.81809	1.66×10^{-10}
PIK3R3	0.322558	2.87×10^{-4}	AGT	-0.57336	0.021398
COL5A3	0.224627	0.016548	RAI14	-0.51113	0.004301
ETV3	0.321939	0.004165	LRRC42	-0.38779	0.0381
ZNF584	0.21347	0.04253	MBOAT2	-0.47655	0.02946
SMYD3	0.172259	0.034218	FOXO6	-1.40311	3.36×10^{-7}
DMGDH	0.172887	0.049819	FZD7	-0.61565	0.034218
POU6F1	0.183904	0.004921	MCAM	-1.07682	1.66×10^{-9}
HPDL	0.352696	0.016066	DAAM2	-0.7124	2.43×10^{-7}
HSPB6	0.469939	2.04×10^{-6}	UNC5B	-0.60098	5.15×10^{-5}
ZBTB7C	0.545789	1.28×10^{-5}	TMEM144	-0.89587	1.16×10^{-9}
SLC30A4	0.269977	1.01×10^{-4}	ERBB3	-0.9177	8.32×10^{-11}
PHF20	0.171941	0.008696	TP53INP2	-1.09019	1.41×10^{-12}
ZNF395	0.189021	0.016658	TYMS	-0.86292	2.16×10^{-10}
TRPS1	0.203594	0.049904	CLCA2	-0.70734	7.41×10^{-7}
PREX1	0.31307	7.48×10^{-5}	PDE8A	-0.59137	7.69×10^{-7}
SPNS2	0.272258	0.024015	MAMDC2	-0.80446	1.48×10^{-5}
NLN	0.279156	0.019506	PRUNE2	-0.65832	2.57×10^{-5}

SDC3	0.252366	0.029695	HACD4	-1.00657	7.15×10^{-5}
INTU	0.279625	0.005451	CYP26A1	-1.39049	2.24×10^{-10}
GEMIN4	0.320309	0.017026	CTSV	-0.9334	4.11×10^{-5}
KCNK12	0.516908	1.32×10^{-4}	ENPP2	-1.28935	4.15×10^{-8}
TTLL6	0.513962	7.15×10^{-6}	REEP3	-1.24276	2.03×10^{-10}
PISD	0.273701	0.010958	FRMD4B	-1.17043	8.16×10^{-7}
PCBD1	0.277118	0.002698	SPSB1	-1.06344	8.95×10^{-8}
MKNK1	0.51745	0.001423	HAPLN2	-1.02737	4.37×10^{-9}
SPATC1L	0.601533	8.03×10^{-6}	ELOVL1	-0.84949	4.44×10^{-7}
CHRD	0.312226	0.003224	HOXD1	-1.35926	2.14×10^{-11}
ENTPD4	0.186061	0.045463	ADAMTS4	-1.32511	6.75×10^{-13}
DNAL4	0.184031	0.027597	ACAN	-1.3214	8.32×10^{-11}
CRIP2	0.229686	0.016117	HHIP	-1.25734	1.02×10^{-12}
PLPPR2	0.230024	0.044254	SLC5A11	-1.57077	1.06×10^{-10}
ATP8B3	0.18137	0.017143	PLEKHH1	-1.61228	1.66×10^{-11}
B4GALT2	0.172646	0.007816	PPP1R14A	-1.59618	6.75×10^{-13}
ZNF618	0.279303	0.004138	MAL	-1.55681	7.50×10^{-13}
PDLIM5	0.253957	0.001088	RASGRP3	-1.65225	1.44×10^{-11}
TMPO	0.175716	0.024541	COL24A1	-0.63925	0.023848
PBXIP1	0.161083	0.038982	OLAH	-0.99939	1.03×10^{-4}
TFDP2	0.337354	5.12×10^{-5}	CBLN1	-0.48194	0.016711
ZNF516	0.349784	2.21×10^{-4}	CRYM	-0.43269	0.020292
HR	0.226678	0.009907	THBS2	-0.91451	1.57×10^{-4}
ATP2B1	0.260426	2.40×10^{-4}	TNC	-0.87566	0.001604
OCA2	0.229271	0.045463	SEMA4C	-0.29797	5.87×10^{-4}
SFRP2	0.321571	0.012458	PLCD3	-0.21531	0.01723
TMEM47	0.733396	2.72×10^{-10}	DGKH	-0.25639	0.020842
TRERF1	0.652962	3.08×10^{-9}	CDK6	-0.25625	0.013632
FAM13A	0.709236	3.86×10^{-12}	FERMT1	-0.4141	4.36×10^{-4}

SERPINE2	0.83003	4.89×10^{-13}	CDC25B	-0.36961	4.36×10^{-4}
TNFRSF1B	0.638672	6.53×10^{-7}	ZYX	-0.39818	2.03×10^{-6}
EOGT	0.829413	1.65×10^{-6}	EXTL1	-0.41415	4.37×10^{-5}
PGAP1	0.367232	0.017143	TTPAL	-0.29397	3.60×10^{-4}
LCORL	0.534405	0.003136	PHLDA3	-0.26544	5.70×10^{-4}
PAX6	0.269385	0.030275	ATF4	-0.3386	5.05×10^{-5}
TDRD6	0.415358	0.004217	PNPLA3	-0.34404	0.001261
DACT2	0.902811	1.06×10^{-10}	STAMBPL1	-0.30973	0.014375
FAT3	0.855573	3.39×10^{-7}	CDR2L	-0.3268	0.003185
FNDC1	0.881946	5.32×10^{-6}	PLXDC1	-0.4026	0.002072
CDH8	0.77032	5.25×10^{-5}	ARC	-0.44508	4.48×10^{-4}
VWC2	0.685795	3.89×10^{-4}	NEU4	-0.3841	0.014443
RSPH9	0.652967	3.62×10^{-4}	NKAIN1	-0.47454	0.003136
KCNN3	0.413642	2.21×10^{-4}	KCNK10	-0.98144	4.25×10^{-10}
ABLIM2	0.466324	6.41×10^{-4}	PTPRD	-0.58072	7.41×10^{-5}
B9D1	0.523311	1.94×10^{-5}	TSKU	-0.33906	0.004212
PALMD	0.330151	0.005517	ID4	-0.27706	0.04696
CHST9	0.282361	1.39×10^{-4}	AQP8	-0.26144	0.038498
THBS1	0.220401	0.004166	SLITRK6	-0.29599	0.016013
DAB1	0.631617	3.02×10^{-10}	GJC2	-0.39542	3.98×10^{-4}
LRRTM1	0.682065	2.40×10^{-5}	CD99	-0.338	0.001238
TMEM100	0.452982	0.010654	RHBDL2	-0.63493	1.75×10^{-7}
EPHX4	1.100916	2.89×10^{-8}	RNF130	-0.48899	1.38×10^{-5}
TWIST1	1.383111	2.25×10^{-7}	OTUD7B	-0.47353	4.27×10^{-7}
EMILIN3	1.412926	3.06×10^{-9}	CPM	-0.3101	0.018185
TESPA1	1.281033	6.83×10^{-11}	CPEB2	-0.43419	4.90×10^{-4}
WFIKKN1	1.049669	1.22×10^{-8}	OPRK1	-0.59186	5.00×10^{-5}
FCN3	1.205192	1.08×10^{-12}	FAT2	-0.57849	2.06×10^{-4}
CBLN4	1.881725	4.98×10^{-18}	TTYH2	-0.72465	5.19×10^{-7}
CDH22	1.8183	4.31×10^{-18}	MYRF	-0.74181	8.94×10^{-9}
FBXO15	1.444453	3.35×10^{-9}	RTKN	-0.62397	6.52×10^{-8}
SV2C	1.232215	1.64×10^{-7}	PLPP2	-0.77316	3.90×10^{-7}

KCTD8	0.442915	0.020853	KLHL5	-0.92053	1.63×10^{-12}
FGF9	0.401325	0.010253	ZFHX3	-0.82521	2.60×10^{-11}
EXTL2	0.38516	0.029893	SMARCD3	-0.42624	0.011387
SCN1A	0.481195	0.004861	PIK3AP1	-0.44712	0.001011
PPARGC1A	0.488449	0.001796	ZNF311	-0.52217	2.34×10^{-5}
LRRK2	0.650388	8.60×10^{-5}	WNT16	-0.54482	2.18×10^{-5}
ACSL6	0.286358	0.015953	CDH11	-0.71023	1.82×10^{-6}
HOMER1	0.365971	0.013076	VAT1L	-0.47328	1.20×10^{-5}
CHRNA1	0.569911	0.008933	F2R	-0.42416	1.28×10^{-4}
ARL9	0.989497	4.01×10^{-6}	WLS	-0.36082	0.003902
RARB	0.894705	2.11×10^{-7}	GRIA1	-0.30718	0.03228
PDZK1	0.762515	0.001627	DSEL	-0.58144	1.26×10^{-8}
RGS22	0.360379	0.035177	PCDH10	-0.45003	2.98×10^{-7}
ANKRD9	0.454848	5.00×10^{-4}	DYRK2	-0.24493	0.048951
VAMP1	0.423392	0.00768	GPD2	-0.51457	1.47×10^{-7}
PRPS2	0.302927	0.038762	ANKRA2	-0.25044	0.027057
PREP	0.365127	0.034352	BCL11A	-0.41851	5.61×10^{-4}
ARNTL	0.387814	0.02725	IQCB1	-0.30312	0.013995
TMEM200C	0.422775	0.005143	NOS1AP	-0.46576	0.004933
PTGFRN	0.469708	0.002388	MTFR1	-0.46584	8.72×10^{-4}
MET	0.604319	0.007894	ENO2	-0.4394	7.59×10^{-4}
RSPO2	1.427513	2.84×10^{-8}	DYNC1LI2	-0.4467	1.06×10^{-4}
CDH7	0.703885	2.54×10^{-4}	ARAP2	-0.45603	0.003952
ADO	0.590369	1.85×10^{-6}	NRG1	-0.50687	4.73×10^{-8}
SESN1	0.573362	5.94×10^{-7}	WT1	-0.33144	3.60×10^{-4}
DCAF6	0.57653	7.19×10^{-5}	EBP	-0.45992	1.20×10^{-5}
ERO1A	0.339621	0.004921	TSPAN18	-0.34579	0.001849
KCNIP2	0.62087	2.07×10^{-7}	KCNA5	-0.44835	2.08×10^{-6}
NLGN1	0.42146	1.28×10^{-4}	NPNT	-0.3154	0.002798
GOLM1	0.514419	3.07×10^{-7}	WDR1	-0.20334	0.025113
GABRB1	0.366788	0.002427	FRMD3	-0.25422	0.039856
TMEM254	0.272882	0.02946	MAPK7	-0.25612	0.018884
LNX1	0.781464	6.31×10^{-6}	TSPAN33	-0.29786	0.001191
CYB5R3	0.723213	3.02×10^{-5}	SOX4	-0.20634	0.011952
AATF	0.444653	0.019506	ACCS	-0.25959	0.020842

CDH12	0.73267	2.27×10^{-4}	ARL8A	-0.22541	0.049904
NLK	0.537862	0.013464	FCHSD1	-0.28945	0.005639
ADGRL2	0.582802	2.39×10^{-5}	NCAM1	-0.2749	0.009131
PENK	0.801266	1.50×10^{-7}	SLC26A2	-0.40333	4.66×10^{-4}
BTG3	0.83107	3.32×10^{-9}	CEP85	-0.43245	6.96×10^{-7}
CHRDL2	1.072271	2.57×10^{-11}	RASEF	-0.31318	0.001742
SLC29A4	0.80928	4.47×10^{-9}	ANKFN1	-0.27416	0.005805
ARHGAP10	0.883055	7.50×10^{-7}	LIX1	-0.25367	0.001999
CAMK4	0.699016	3.00×10^{-7}	ABHD17B	-0.28214	8.00×10^{-4}
PTPRF	1.063687	1.24×10^{-12}	SLCO4A1	-0.17868	0.040565
FEM1C	1.077473	4.83×10^{-6}	PLCB4	-0.26546	0.007098
DACT1	0.539383	2.76×10^{-5}	IDH1	-0.23485	0.001627
DCLK3	0.953681	1.97×10^{-5}	CNTN4	-0.21079	0.006107
SYT17	0.559444	0.032313	OPRM1	-0.32483	1.06×10^{-4}
DIAPH2	1.109426	6.90×10^{-6}	NOL3	-0.22954	0.002545
TAF7L	1.303102	5.19×10^{-9}	TRDN	-0.32753	4.33×10^{-6}
NECAB2	1.616635	1.24×10^{-12}	PRMT2	-0.31802	7.51×10^{-6}
PVALB	0.767795	0.009884	DCBLD2	-0.2492	0.02839
TRIM37	-0.15822	0.038726	CCNE2	-0.30709	0.004158
ACSS2	-0.17631	0.033896	RECK	-0.33027	0.006515
NFE2L2	-0.27772	6.82×10^{-4}	SLC24A2	-0.39767	0.003539
DENND5A	-0.29465	0.007419	DCLRE1C	-0.53892	2.76×10^{-6}
CHD1L	-0.25224	0.018293	KLF7	-0.45696	2.61×10^{-5}
TFRC	-0.28778	0.013544	CAMK2G	-0.53418	1.02×10^{-6}
VAV3	-0.35756	4.11×10^{-5}	ACVR2A	-0.32339	0.005816
GALNT12	-0.21197	0.029134	SUMF1	-0.30228	0.020163
FBXO40	-0.21985	0.034352	IQSEC1	-0.21166	0.046835

Note: Genes are sorted (top to bottom) in the order of the heatmap columns (left to right) from Figure 5d, right panel.

SI References

[1] P. Barttfeld, L. Uhrig, J. D. Sitt, M. Sigman, B. Jarraya, S. Dehaene, *Proc. Natl. Acad. Sci. USA* **2015**, *112* (3), 887.
